# Regulation of Hemolysin Expression and Virulence of *Staphylococcus aureus* by a Serine/Threonine Kinase and Phosphatase

**DOI:** 10.1371/journal.pone.0011071

**Published:** 2010-06-11

**Authors:** Kellie Burnside, Annalisa Lembo, Melissa de los Reyes, Anton Iliuk, Nguyen-Thao BinhTran, James E. Connelly, Wan-Jung Lin, Byron Z. Schmidt, Anthony R. Richardson, Ferric C. Fang, Weiguo Andy Tao, Lakshmi Rajagopal

**Affiliations:** 1 Department of Pediatric Infectious Diseases, University of Washington and Seattle Children's Hospital Research Institute, Seattle, Washington, United States of America; 2 Department of Biochemistry, Purdue University, West Lafayette, Indiana, United States of America; 3 Departments of Laboratory Medicine and Microbiology, University of Washington, Seattle, Washington, United States of America; Cairo University, Egypt

## Abstract

Exotoxins, including the hemolysins known as the alpha (α) and beta (β) toxins, play an important role in the pathogenesis of *Staphylococcus aureus* infections. A random transposon library was screened for *S. aureus* mutants exhibiting altered hemolysin expression compared to wild type. Transposon insertions in 72 genes resulting in increased or decreased hemolysin expression were identified. Mutations inactivating a putative cyclic di-GMP synthetase and a serine/threonine phosphatase (Stp1) were found to reduce hemolysin expression, and mutations in genes encoding a two component regulator PhoR, LysR family transcriptional regulator, purine biosynthetic enzymes and a serine/threonine kinase (Stk1) increased expression. Transcription of the *hla* gene encoding α toxin was decreased in a Δ*stp1* mutant strain and increased in a Δ*stk1* strain. Microarray analysis of a Δ*stk1* mutant revealed increased transcription of additional exotoxins. A Δ*stp1* strain is severely attenuated for virulence in mice and elicits less inflammation and IL-6 production than the Δ*stk1* strain. *In vivo* phosphopeptide enrichment and mass spectrometric analysis revealed that threonine phosphorylated peptides corresponding to Stk1, DNA binding histone like protein (HU), serine-aspartate rich fibrinogen/bone sialoprotein binding protein (SdrE) and a hypothetical protein (NWMN_1123) were present in the wild type and not in the Δ*stk1* mutant. Collectively, these studies suggest that Stk1 mediated phosphorylation of HU, SrdE and NWMN_1123 affects *S. aureus* gene expression and virulence.

## Introduction

Invasive bacterial infections remain a significant cause of morbidity and mortality in humans [1]. *Staphylococcus aureus* is among the most common human pathogens. Although 20% of the population are asymptomatically colonized with *S. aureus* in the skin, upper respiratory or gastrointestinal tracts, *S. aureus* is also the leading cause of invasive infections in both community and in healthcare settings [2], [3], [4]. Clinical manifestations of *S. aureus* range from superficial skin infections to severe or deep-seated infections such as pneumonia, bacteremia, osteomyelitis, endocarditis and toxic shock [5].

A number of virulence factors that include hemolysins, exotoxins, leukocidins, superantigens, capsule and secreted enzymes allow *S. aureus* to overcome host defenses (for recent reviews, see [6], [7]). *S. aureus* lysis of red blood cells is primarily mediated by the hemolysins known as alpha (α), beta (β) and delta (δ) toxins. The α toxin encoded by the *hla* gene is important for *S. aureus* pneumonia, sepsis, septic arthritis, brain abscess and corneal infections [8], [9], [10], [11], [12], [13]. This 33kDa pore forming toxin is secreted by majority of *S. aureus* clinical isolates and is active against a wide range of mammalian cells, with especially marked activity against rabbit erythrocytes [14], [15]. In addition to its pore forming ability, α toxin induces the release of cytokines and chemokines such as IL-6, IL-1β, IL-1α, IL-8, TNF-α, KC and MIP-2 [9], [16], [17], [18], [19], [20]. Immunization with inactive α toxin was recently shown to protect mice against lethal *S. aureus* pneumonia [21], [22], [23]. These observations emphasize the importance of α toxin in *S. aureus* infections.

Certain strains of *S. aureus* also secrete beta (β) toxin, a 35kDa sphingomyelinase encoded by the *hlb* gene [24], [25], [26]. In contrast to α toxin, β toxin is highly hemolytic for sheep but not for rabbit erythrocytes [27]. Hemolytic activity of β toxin is enhanced after incubation at temperatures below 10°C, hence this toxin is often referred to as the ‘hot-cold’ hemolysin [24], [27]. The importance of β toxin has been demonstrated in *S. aureus* infections of the human lung and cornea, and an ability to inhibit the ciliary activity of nasal epithelial cells has been described [28], [29], [30]. The presence of a cleavable signal sequence in the N-terminal region of α and β toxins suggests that they are secreted by the general secretory (sec) pathway (for a review, see [31]). Although not much is known about the Sec pathway in *S. aureus*, this pathway has been extensively characterized in *B. subtilis* and *E. coli* [32], [33].

Delta (δ) hemolysin or toxin is a 26 amino acid peptide encoded by the *hld* gene [34], [35]. This toxin is produced by 97% of *S. aureus* isolates and lyses erythrocytes, a variety of mammalian cells and sub-cellular structures such as membrane bound organelles, spheroplasts and protoplasts [36], [37]. In contrast to the α and β toxins, δ toxin does not possess a cleavable signal sequence [27], and its mechanism of secretion is not completely understood. Because the structural gene for δ toxin is encoded within the RNA molecule (RNAIII) that activates transcription of a number of virulence factors such as α toxin, enterotoxins, and toxic shock syndrome toxin, and represses the transcription of cell surface proteins such as protein A, the precise contribution of δ toxin to *S. aureus* virulence is not known [38], [39].

Despite the importance of *S. aureus* as a pathogen, our understanding of the molecular events that enable the organism to make the transition from a commensal state into an invasive pathogen is limited. Mechanisms that facilitate *S. aureus* infiltration into deeper tissues with subsequent dissemination and invasion of multiple organs remain to be completely understood. However, the diverse array of host niches encountered by *S. aureus* suggests that the pathogen efficiently adapts to changing environments to survive and establish successful infections. Pathogenic bacteria commonly utilize signaling systems to adapt, survive and invade a variety of host niches [40], [41], [42]. Signaling is primarily achieved through reversible phosphorylation of proteins, and these modifications are the most critical of the 200 different types of post-translational modifications [43], [44]. A number of two-component systems (TCS) and one component transcriptional regulators regulate the transcription of *S. aureus* virulence genes [45], [46]. These include the complex accessory gene regulatory system (*agrABCD*) that up-regulates the expression of superantigens, cytotoxins, and secreted enzymes, and represses the transcription of cell-wall proteins such as protein A and fibronectin binding proteins [38], [45]. Other TCS known as SaeR/SaeS and ArlR/ArlS also regulate the transcription of α (*hla*) and β (*hlb*) toxins in *S aureus* [47], [48], [49], [50]. The staphylococcal accessory regulator known as SarA binds to conserved promoter regions known as ‘Sar boxes’ for transcriptional regulation. Sar homologues such as SarT and Rot are also described to repress transcription of *hla* [51], [52], [53], [54], [55].

In this study, we screened a random transposon library for mutants that showed either an increase or decrease in hemolysin expression compared to wild type (WT). Our studies identified 72 genes that affect hemolysin expression in *S. aureus* and include signaling enzymes commonly found in eukaryotes. We further demonstrate that these eukaryotic-like signaling enzymes affect α toxin (*hla*) transcription and *S. aureus* virulence.

## Results

### Identification of genes that regulate hemolytic activity of *S. aureus*

Previous studies have established that hemolysins are important for infections caused by *S. aureus*. To identify novel factors that regulate hemolysin expression in *S. aureus*, we screened a transposon insertion library for mutants that showed either an increase or decrease in hemolytic activity compared to the wild type (WT) strain. The transposon library was obtained as a kind gift from Chiron Corporation/Novartis Vaccines (CMCC #51963, patent WO/2004/018624). This library consists of 6,725 individual, random transposon Tn5EZ mutants for which the insertion sites of the transposons in the *S. aureus* genome are known (see patent WO/2004/018624 entitled ‘Random transposon insertion in *Staphylococcus aureus* and use thereof to identify essential genes’ at http://www.wipo.int/pctdb/en/wo.jsp?WO=2004%2F018624&IA=WO2004%2F018624&DISPLAY=DESC). The Tn5EZ mutants were derived from *S. aureus* RN4220, and all 6,725 mutants are contained on individual, 96-well microtiter plates. Although RN4220 has the genes encoding α (*hla*), β (*hlb*), and δ (*hld*) toxins, an extra adenine residue in the 3′ end of the gene encoding *agrA* decreases α and δ toxin expression (for details, see[56]). As expression of β toxin is not tightly regulated by *agr* [56], hemolytic activity due to this toxin is seen in RN4220. We hypothesized that screening the transposon library for mutants with altered hemolytic activity would enable us to identify genes that affect α and δ toxin expression despite decreased *agr* regulation, and also identify genes that regulate β toxin expression.

**Figure 1:**
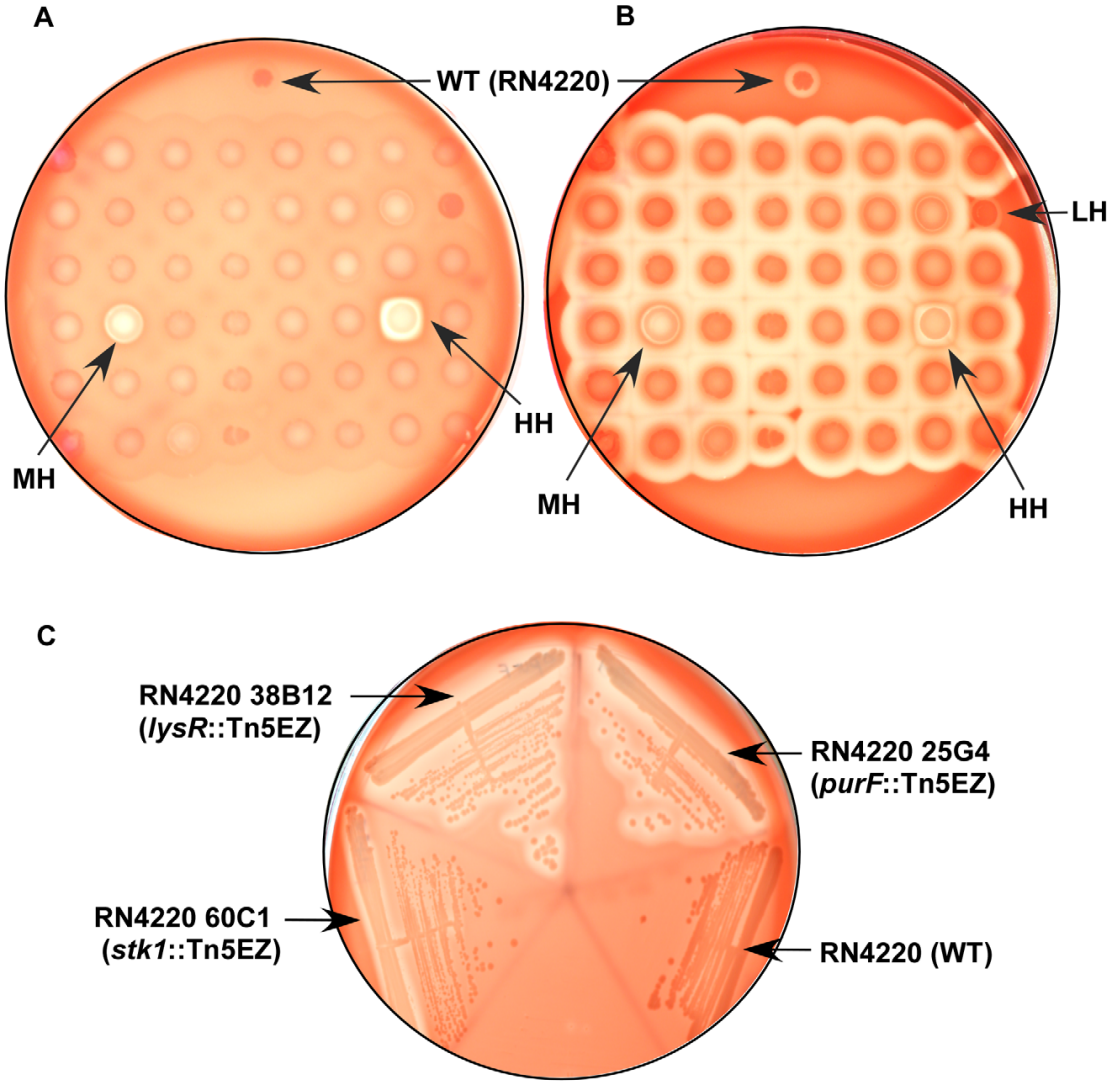
*S. aureus* transposon mutants showing increased or decreased hemolysis. Hemolytic activity is represented by the zone of clearing around the colonies on the sheep blood agar plates. (**A**) and (**B**) represent the same sheep blood agar (SBA) plate. Forty-eight random TN5EZ mutants of *S. aureus* were stamped on a SBA plate and incubated O/N at 37°C. The plate was then incubated at 4°C O/N. (**A**) shows hemolysin activity after the 37°C incubation and (**B**) represents the same plate after the 4°C incubation. The wild type strain (WT, RN4220) was spotted as a control. Note that a few mutants showed hyper-hemolysis (HH), moderately hyper-hemolysis (MH) or lower hemolysis (LH) compared to WT in both A and B. These mutants were chosen for further analysis (Table 1–4). (**C**) Strains with transposon TN5EZ insertions in SA2555 (putative *lysR* family regulator), *purF* and *stk1* show a marked increase in hemolytic activity compared to WT RN4220 on rabbit blood agar (RBA).

To identify mutants that showed altered hemolytic activity, we used a 48-well prong to transfer individual colonies onto sheep blood agar plates (SBA). These plates were incubated at 37°C overnight. Subsequently, the plates were transferred to 4°C overnight, and colonies that exhibited increased or decreased hemolytic activity after incubation at 37°C and 4°C were identified by visual screening (see Fig. 1, A and B for a representative plate). Analysis of the selected mutants on SBA was repeated to confirm the reproducibility of the hemolytic phenotype. Our studies indicate that transposon insertions in (a) 13 genes abolished hemolytic activity (No Hemolysis, NH), (b) 25 genes conferred decreased hemolysis (Less Hemolysis, LH), (c) 26 genes caused a moderate increase in hemolysis (Moderately hyper-hemolysis, MH), and (d) 8 genes conferred hyper-hemolysis (Hyper hemolysis, HH) when compared to the WT and most other Tn5EZ mutants on the same SBA plate (Table 1–4, Fig. 1 A and B).

**Table 1:** TN5EZ insertions that abolished *S. aureus* hemolysin activity.

# of mutants*	Hemolysis	Gene/Locus	Function or predicted function
2	NH	SA0014	Modified GGDEF guanylate cyclase/cyclic diGMP synthetase
1	NH	SA0101	Ornithine cyclodeaminase
2	NH	SA0164	Gramicidin S synthetase 2 – Non-ribosomal peptide synthetase
1	NH	SA0403	Transcriptional antiterminator, BglG family
4	NH	***saeR***	Two-component regulator SaeR
1	NH	*mnhE*	Na/H antiporter, MnhE component
1	NH	*fmt*	Methionyl-tRNA formyltransferase
1	NH	*infB*	Translation initiation factor IF-2
1	NH	*dapA*	Dihydrodipicolinate synthase - aspartate family
2	NH	***hlb***	Phospholipase C, Fatty acid and lipid metabolism
2	NH	SA2072	ATP-dependent RNA helicase, DEAD/DEAH box family
1	NH	*atpF*	ATP synthase F0, B subunit
1	NH	SA0495	Hypothetical protein

*NH represents no hemolysis compared to WT and most other Tn5EZ mutants on sheep blood agar (also see Fig. 1). # of mutants represents the number of individual transposon insertions in the same gene or loci that alter hemolysin activity.

**Table 2 pone-0011071-t002:** TN5EZ insertions that reduced *S. aureus* hemolysin activity.

# of mutants*	Hemolysis	Gene/Locus	Function or predicted function
1	LH	SA0466	Membrane protein, putative
1	LH	*bioD*	Dethiobiotin synthase - Biosynthesis of cofactors
2	LH	SA0555	Peptidase, M41 - Degradation of proteins, peptides,
1	LH	SA0635	Lipoate-protein ligase A - Protein modification
1	LH	SA0762	Hemolysin, putative -
3	LH	*gcvH*	Glycine cleavage system H protein - energy metabolism
1	LH	SA0958	General stress protein 13
1	LH	SA1049	1,4-dihydroxy-2-naphthoate octaprenyltransferase, putative
1	LH	SA1220	Fibronectin-binding protein A-related
2	LH	SA1294	Metallo-beta-lactamase superfamily protein
1	LH	*glpD*	Aerobic glycerol-3-phosphate dehydrogenase
1	LH	SA1563	2-oxoisovalerate dehydrogenase, lipoamide dehydrogenase,
1	LH	*secDF*	Protein-secretion, membrane protein, SecDF family
1	LH	SA1751	DHH family protein, putative
2	LH	*ackA*	Acetate kinase - fermentation
1	LH	*ccpA*	Catabolite control protein - DNA interactions
1	LH	***agrA***	Accessory gene regulator protein A
1	LH	SA2027	Hypothetical protein - not conserved
9	LH	*atpDGA*	ATP synthase F1, beta, gamma and alpha subunits -
1	LH	SA2271	Molybdenum ABC transporter, permease protein - cations
1	LH	SA2375	Transporter - unknown substrate
1	LH	SA2534	NAD(P)H-flavin oxidoreductase
1	LH	SA2572	Cation-transporting atpase, E1-E2 family
1	LH	SA2624	Acetyl-CoA synthetase, putative
3	LH	SA2735	Chromosome partioning protein, ParB family

*LH represents lower hemolysis compared to WT and most other Tn5EZ mutants on sheep blood agar (also see Fig. 1).

**Table 3 pone-0011071-t003:** TN5EZ insertions that increased *S. aureus* hemolysin activity.

# of mutants*	Hemolysis	Gene/Locus	Function or predicted function
2	MH	*gyrA*	DNA gyrase, A subunit
9	MH	SA0007	YjeF-related protein, C-terminus - unknown function
3	MH	*hutH*	Histidine ammonia-lyase
1	MH	SA0216	Hypothetical protein
1	MH	SA0237	Alcohol dehydrogenase, zinc-containing
1	MH	SA0311	Sodium:solute symporter - Transport and binding proteins
1	MH	*geh*	Lipase precursor, interruption - disrupted reading frame
6	MH	*ksgA*	Dimethyladenosine transferase
1	MH	SA0713	Hypothetical protein - not conserved
1	MH	*fruB*	1-phosphofructokinase - Glycolysis/gluconeogenesis
1	MH	SA1021	Multidrug-efflux transporter, putative
5	MH	*purL*	Phosphoribosylformylglycinamidine synthase II
1	MH	SA1096	TRK system potassium uptake protein TrkA, putative - cations
1	MH	*pdhD*	Pyruvate dehydrogenase, E3 component,
2	MH	*stk1*	Protein kinase - regulatory functions - protein interactions
1	MH	SA1309	Pyruvate ferredoxin oxidoreductase, beta subunit,
1	MH	SA1472	Pathogenicity protein, putative
2	MH	*era*	GTP-binding protein Era - small molecule interactions
1	MH	SA1740	Two component DNA-binding regulator, PhoP
1	MH	***rot***	Virulence factor regulator protein
3	MH	*fumC*	Fumarate hydratase, class II - TCA cycle
2	MH	*pcrA*	PcrA protein - DNA replication, recombination, and repair
1	MH	*hutU*	Urocanate hydratase - Amino acids and amines
1	MH	SA2308	Transcriptional regulator, RpiR family
9	MH	SA2737	Glucose inhibited division protein A
12	MH	*thdF*	Thiophene and furan oxidation protein ThdF - detoxification

MH represents moderately hyper-hemolysis compared to WT and other Tn5EZ mutants.

**Table 4:** TN5EZ insertions that significantly increased *S. aureus* hemolysin activity.

# of mutants*	Hemolysis	Gene/Locus	Function or predicted function
3	HH	SA0704	Iron compound ABC transporter, ATP-binding protein
5	HH	*purF*	Amidophosphoribosyltransferase - Purine biosynthesis
2	HH	*ptsl*	Phosphoenolpyruvate-protein phosphotransferase
2	HH	*pnp*	Polyribonucleotide nucleotidyltransferase - Degradation of RNA
1	HH	SA2555	Transcriptional regulator, LysR family - DNA interactions
1	HH	SA2113	Transcription termination factor Rho - transcription factors
1	HH	SA2348	QacA subfamily/drug transporter, putative
1	HH	SA1281	Membrane-associated zinc metalloprotease,

*HH represents hyper-hemolysis compared to WT and most other Tn5EZ mutants on sheep blood agar (also see Fig. 1).

As expected, transposon insertions in the *hlb* gene encoding β toxin, and a few known hemolysin activators such as *agrA* [38], [45], *saeR* [47], [48] and *ccpA* [57] decreased and/or abolished hemolytic activity in RN4220 (Table 1 and 2). Likewise, a Tn5EZ insertion in the previously described hemolysin repressor *rot* increased hemolytic activity (Table 3 and [55]). As the transposon library did not contain insertions in the structural genes for α toxin (*hla*), δ toxin (*hld*) and other known hemolysin regulators such as *sarART* and *agrBC* [45], these genes were not identified in the above analysis. A transposon insertion in the gene encoding acetate kinase down-regulated hemolysin expression; this may be due to decreased synthesis of acetyl phosphate, an intracellular signal for phosphorylation of two component response regulators (for a review, see [58]). Taken together, the above findings validate our screen for altered hemolysin expression in *S. aureus*. Importantly, our studies also identified a number of novel genes that regulate hemolysin expression of *S. aureus* (Table 1–4). Transposon insertions in the two component regulator PhoR, purine biosynthetic gene *purF*, a gene predicted to encode a transcriptional regulator of the LysR family (SA2555), and the serine/threonine kinase gene *stk1* increased the hemolytic phenotype (see Table 3 and 4). As the increase in hemolytic activity of these mutants was observed at 37°C, we hypothesized that this may be due to an increase in α and/or δ toxin, as β toxin-mediated lysis of red blood cells is primarily seen after incubation at temperatures below 10°C [24], [27]. Therefore, we compared hemolytic activity of these strains on rabbit blood agar (RBA), as α toxin demonstrates a >1000 fold increase in lysis of rabbit erythrocytes compared to sheep erythrocytes, whereas β toxin does not lyse rabbit erythrocytes [59]. Lysis of rabbit red blood cells (rRBC) was observed in strains with Tn5EZ insertions in SA2555, *purF* and *stk1*, in contrast to WT RN4220 (Fig. 1C), suggesting that these loci repress α toxin expression. As expected, the NH and LH strains did not demonstrate any difference in hemolysis on RBA compared to WT RN4220 (data not shown).

### A serine/threonine kinase and phosphatase regulate hemolysin expression in *S. aureus*

**Figure 2:**
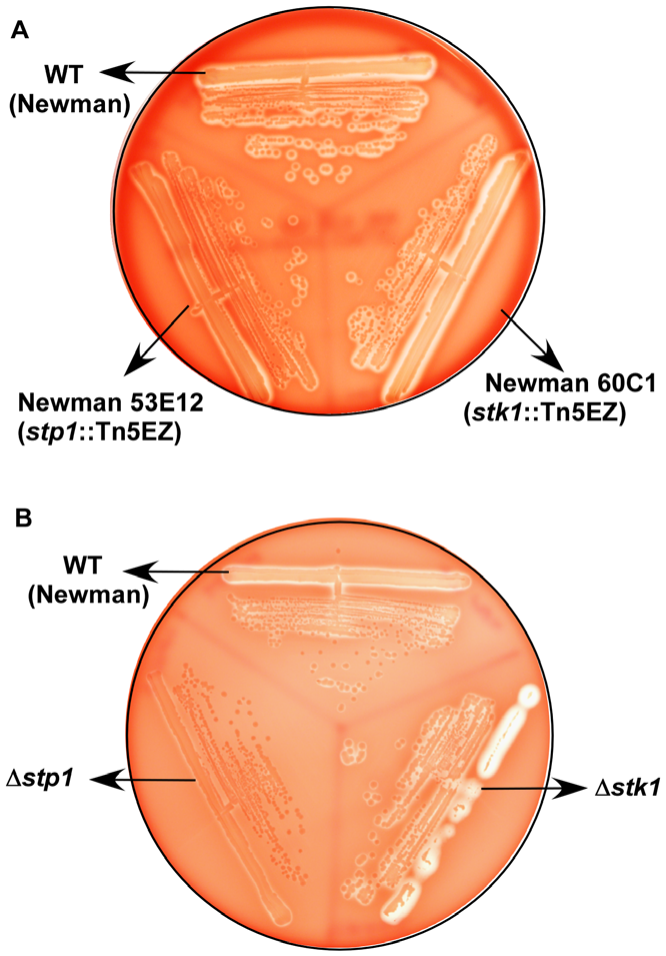
A serine/threonine phosphatase and kinase oppositely regulate hemolytic activity of *S. aureus*. (**A**) A transposon (Tn5EZ) insertion in *stp1* (53E12) decreased hemolytic activity whereas the strain with the Tn5EZ insertion in *stk1* (60C1) increased hemolytic activity compared to the isogenic WT Newman. (**B**) shows hemolytic activity of Newman WT, Δ*stp1* and Δ*stk1* strains on sheep blood agar. Note that hemolytic activity is decreased in the Δ*stp1* strain and increased in a Δ*stk1* mutant compared to WT Newman.

The transposon screen indicated that an insertion in *stk1* confers an increase in *S. aureus* hemolysin activity (Table 3). Previous studies from our laboratory have identified that homologues of *stk1* regulate hemolysin expression in the gram-positive human pathogen group B streptococcus [60], [61]. Therefore, we were interested in characterizing the role of Stk1 and its cognate serine/threonine phosphatase Stp1 in the regulation of hemolysin expression and virulence of *S. aureus*. To confirm that *stk1* and *stp1* regulate α toxin expression, phage φ11 [62] was used to transduce the Tn5EZ insertions in *stk1* (Table 3) and *stp1* from the transposon library in RN4220 into the virulent strain of *S. aureus* known as Newman. *S. aureus* Newman is proficient in expression of α and δ toxin but deficient for β toxin due to the insertion of a prophage in *hlb* [63]. A comparison of hemolytic activity of these strains indicated that a Tn5EZ insertion in *stp1* (Newman 53E12) decreased hemolytic activity, whereas a Tn5EZ insertion in *stk1* (Newman 60C1) increased hemolytic activity compared to WT Newman (Fig. 2A).

To further establish the role of Stp1 and Stk1 on hemolysin expression and to examine their role in virulence of *S. aureus*, allelic exchange mutants (AEM) were constructed. To this end, the coding sequences of *stp1* and *stk1* were replaced with genes that conferred either chloramphenicol (Cm, [64]) or kanamycin resistance (Kn, [65]) in *S. aureus* RN4220 and subsequently transduced into *S. aureus* Newman (see Methods for details). We then compared the hemolytic activity of WT to isogenic Δ*stp1* and Δ*stk1* strains. Consistent with the transposon mutants (Fig. 2A), hemolytic activity was lower in a Δ*stp1* mutant constructed by allelic exchange and higher in the Δ*stk1* mutant, compared to WT Newman (Fig. 2B). Although changes in hemolysin expression are often observed in *S. aureus* due to spontaneous nonsense mutations in the *agr* regulatory system [56], [66], a comparison of the DNA sequence of the *agr* locus (*agrABCD*) between WT Newman and the isogenic Δ*stp1* and Δ*stk1* strains did not indicate the presence of any spontaneous *agr* mutations (data not shown). Similar changes in hemolysin expression were also observed in Δ*stk1* and Δ*stp1* mutants derived from WT *S. aureus* RN6390 (data not shown). Taken together, these data indicate that Stp1 and Stk1 oppositely regulate hemolytic activity of *S. aureus*.

### Role of Stp1 and Stk1 in transcription of *hla*

**Figure 3:**
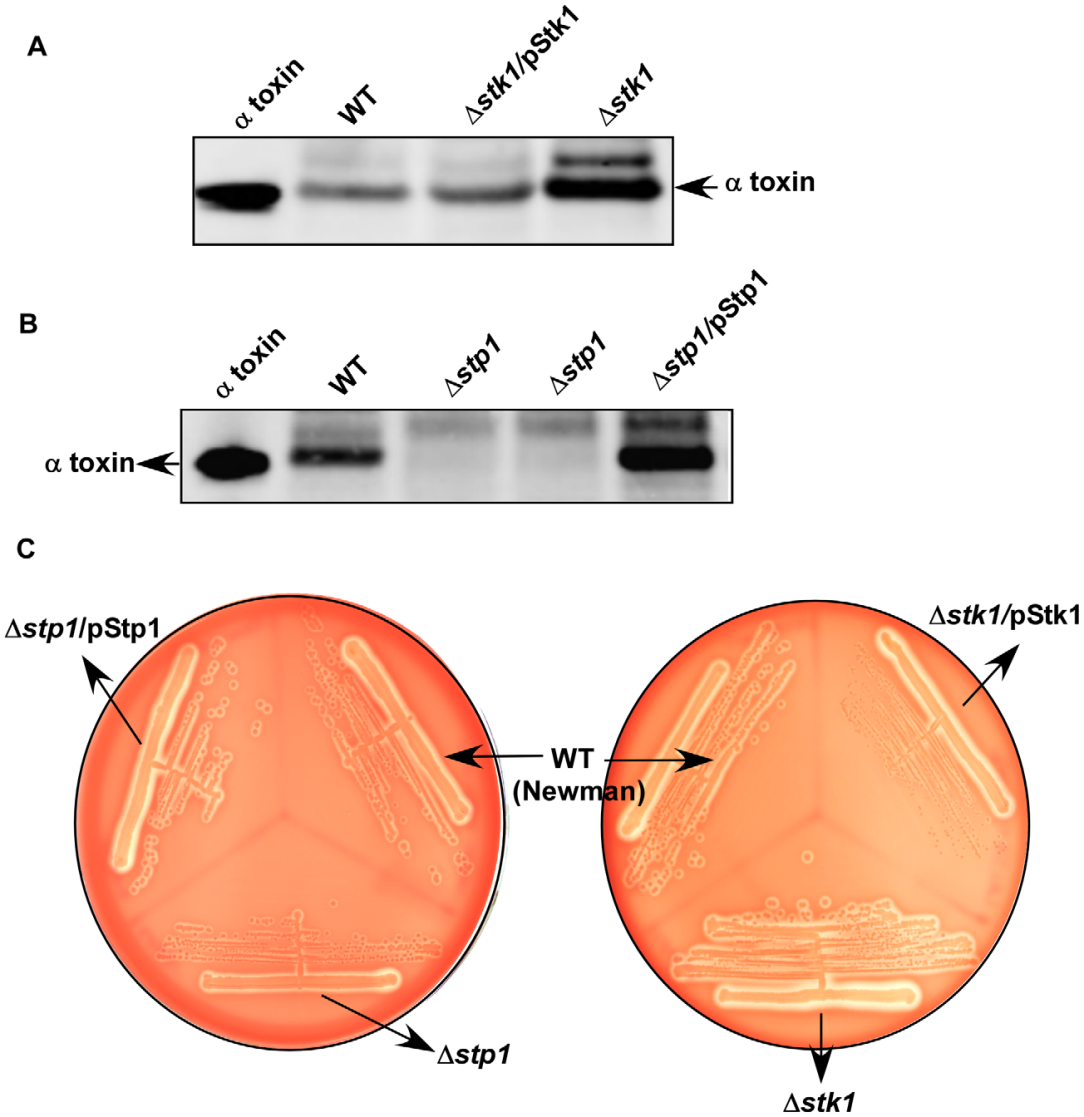
Extracellular α toxin is decreased in a Δ*stp1* mutant and increased in a Δ*stk1* mutant compared to WT Newman. (A) and (B) Quantitative western blots for α toxin were performed on equal amounts of extracellular protein from *S. aureus* WT (Newman), Δ*stp1*, Δ*stk1* and the complemented strains. As a control, 1µg purified α toxin (Sigma Chemical Co, USA) was included in these assays. Secreted or extracellular α toxin is higher in a Δ*stk1* mutant and lower in a Δ*stp1* mutant compared to WT Newman. Two independent Δ*stp1* cultures were examined in Panel B. Note that α toxin is restored to WT levels in the complemented Δ*stk1* (Δ*stk1*/pStk1) and Δ*stp1* strains (Δ*stp1*/pStp1), respectively. (**C**) shows hemolytic activity of complemented Δ*stp1* and Δ*stk1* strains. Note that decreased hemolytic activity in a Δ*stp1* mutant is restored to WT levels in the complemented Δ*stp1/*pStp1 strain. Likewise, increased hemolytic activity in a Δ*stk1* mutant is restored to WT levels in the complemented Δ*stp/*pStk1 strain.

We next examined whether the absence of Stp1 or Stk1 affected the transcription of the gene encoding α toxin. To test this possibility, RNA was isolated from three independent biological replicates of WT *S. aureus* Newman and isogenic Δ*stp1* and Δ*stk1* strains grown in tryptic soy broth (TSB) to an optical density (OD_600_) of 0.6 (exponential phase) or ∼3.0 (post-exponential phase), and quantitative real time PCR (qRT-PCR) was performed as described [67]. The housekeeping gene *rpoD* was used to normalize transcription of *hla* and *hld* by the comparative C_T_ method [68]. In these studies, we also compared transcription of the gene encoding δ toxin (*hld*), the Agr-repressed gene encoding Protein A (*spa*) and *agrA*. We observed that transcription of *hla* was approximately 2-fold lower in the Δ*stp1* mutant and significantly higher (6.4 fold) in the Δ*stk1* mutant compared to WT, but only during post-exponential phase growth (Table 5, P<0.05, student's t test). Under these conditions, transcription of *hld*, *spa* and *agrA* were not significantly different between WT, Δ*stp1* and Δ*stk1* mutants (Table 5). Given that AgrA positively regulates transcription of *hla* and *hld*, and negatively regulates *spa* transcription during post-exponential phase growth of *S. aureus*, our results suggest that Stp1 and Stk1 regulation of α toxin transcription is independent of global Agr regulation. To confirm that the change in *hla* transcription in Δ*stp1* and Δ*stk1* correlated with altered α toxin protein levels, we performed quantitative western blots as described ([21], also see Methods). Four independent experiments indicated that extracellular or secreted α toxin was 1.7–2 fold higher in the Δ*stk1* strain compared to WT *S. aureus* Newman (Fig. 3A). Decreased α toxin levels were observed in the Δ*stp1* strain compared to WT Newman (Fig. 3B). However, intracellular α toxin levels in Δ*stp1* and Δ*stk1* mutants were not consistently different from that of WT (data not shown).

**Table 5:** Transcription of *hla* is decreased in S. *aureus* Δ*stp1* and increased in Δ*stk1* mutants.

Relative Gene Expression#				
	*hla* (α toxin)	*hld* (δ toxin)	*agrA*	*spa*
Δ*stp1*	0.31±0.15*	0.78±0.53	0.55±0.18	2.0±1.92
Δ*stk1*	6.42±1.98*	1.33±0.09	1.19±0.32	1.39±0.47
Δ*stp1*/pStp1	1.5±0.5	NT	NT	NT
Δ*stk1*/pStk1	2.0±0.5	NT	NT	NT

# qRT-PCR was performed as described in Methods. Gene expression is denoted as fold difference relative to the WT *S. aureus* strain Newman. Standard deviation is indicated. NT indicates not tested.

*indicates statistically significant, *P* values<0.05, 2 tailed t test.

To confirm that the change in hemolytic activity of the Δ*stp1* and Δ*stk1* strains was due to the absence of these genes, we performed complementation studies. The genes encoding *stp1* and *stk1* were PCR-amplified and cloned into the complementation vector pDCerm to generate pStp1 and pStk1, respectively (Table S1). The complementing plasmids pStp1 and pStk1, and the vector pDCerm, were electroporated into the RN4220 Δ*stp1* and Δ*stk1* strains. Subsequently, using φ-11, these plasmids were transduced into Newman Δ*stp1* and Δ*stk1* strains. qRT-PCR and quantitative western blots confirmed that α toxin was restored to WT levels in both the Δ*stp1* and Δ*stk1* strains containing the complementing plasmids (Table 5, Fig. 3A & B). Hemolytic activity was restored to WT levels in both the Δ*stp1* and Δ*stk1* strains containing the complementing plasmids (see Δ*stp1*/pStp1 and Δ*stk1*/pStk1 in Fig. 3C), in contrast to those containing only the vector pDCerm. These data confirm that Stp1 and Stk1 regulate expression of α toxin in *S. aureus*.

### Microarray analysis

The change in *hla* transcription observed in the Δ*stp1* and the Δ*stk1* strains (Table 5) prompted us to examine global changes in gene expression during post-exponential phase growth (OD_600_∼3.0). Transcriptional profiling analysis was performed on RNA isolated from WT Newman and isogenic Δ*stp1* and Δ*stk1* mutants, and the results are shown in Table S2. These results confirmed that *stk1* expression was similar to WT in the Δ*stp1* strain but was 200-fold lower in the Δ*stk1* mutant (Table S2). Similarly, *stp1* expression was 100-fold decreased in the Δ*stp1* strain but similar to WT in the Δ*stk1* mutant (Table S2). A comparison of gene expression between WT and Δ*stp1* or Δ*stk1* indicated that 32 genes were up-regulated and 43 genes were down-regulated in the Δ*stk1* strain, whereas 84 genes were up-regulated and 55 genes were down-regulated in the Δ*stp1* strain (Table S2). As expected, transcription of α toxin was decreased in the Δ*stp1* mutant and increased in the Δ*stk1* mutant. Interestingly, transcription of a number of other exotoxins including LukF and LukS were increased in a Δ*stk1* background (2.0–4.8 fold, Table S2) whereas transcription levels of these genes was similar to the WT in the Δ*stp1* strain. In contrast, transcription of genes encoding capsular polysaccharide biosynthetic enzymes was 2.0–2.5 fold increased in the Δ*stp1* mutant and decreased in the Δ*stk1* mutant. A comparison of genes that regulate hemolysin activity (Table 1–4) to those observed in the microarray (Table S2) indicated that genes important for hemolysin activity, such as FruB, SACOL0495 and SACOL2072 (see ** in Table S2), showed decreased expression in the Δ*stp1* mutant. Conversely, *dapA* encoding dihydrodipicolinate synthase, that is also important for hemolysin expression (Table 1), showed a modest increase in transcription only in the Δ*stp1* mutant (see * in Table S2). Surprisingly, decreased transcription of genes important for hemolytic activity, i.e. FruB and the transcriptional anti-terminator SACOL0403 (Table 3), were observed in the Δ*stk1* mutant (see ^#^ in Table S2). Whether altered transcription of these genes contributes to the change in hemolytic activity in Δ*stp1* and Δ*stk1* (Fig. 2) mutant strains has yet to be established.

### Stp1 is essential for *S. aureus* virulence

**Figure 4:**
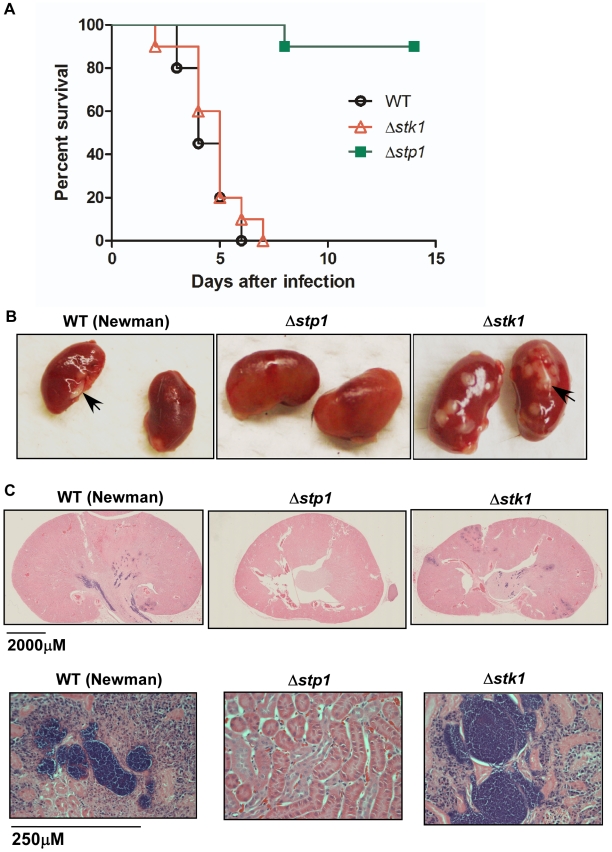
Stp1 is important for virulence of *S. aureus*. (**A**) Ten, six week-old female C57BL/6J mice were intravenously injected with 2–5×10^7^ CFU of *S. aureus* WT, Δ*stp1* or Δ*stk1* strains. As a control, ten mice were inoculated in parallel with PBS. Kaplan-Meier survival curves show the percent survival of mice after the infection. Note that 100% of mice infected with the WT and Δ*stk1* strains succumbed to infection, in contrast to only 10% of mice infected with the Δ*stp1* mutant. Survival of mice infected with the Δ*stp1* mutant was significantly different from that of the WT and the Δ*stk1* mutant (*P* value<0.0001, log rank test). However, mice infected with the Δ*stk1* mutant did not show a significant difference in survival compared to the WT (*P* value = 0.44, log rank test). (**B**) Kidneys harvested from the mice that were moribund or at the end of the experiment. Arrows represent abscesses that were observed on the infected kidneys. Note that a large number of abscesses are seen in the kidneys of mice infected with the Δ*stk1* mutant in comparison to those infected with the Δ*stp1* strain. One representative from ten infected kidney pairs after inoculation with the *S. aureus* strain is shown. (**C**) Histological examination of infected renal tissue was performed at 5 days post-infection. Kidneys were fixed in formalin, embedded in paraffin, thin-sectioned, stained with hematoxylin and eosin (H&E), and examined by microscopy as described previously [69], [97]. Abscesses and necrotic tissue are observed in kidneys of mice infected with WT and Δ*stk1* strains but not with the Δ*stp1* mutant.

The virulence of Δ*stp1* and Δ*stk1* mutants was compared with WT using the mouse sepsis model of infection described previously [69]. Six week-old female C57BL/6J mice (*n* = 10) were inoculated intravenously with 2–5×10^7^ colony-forming units (CFU) of WT, Δ*stp1* or Δ*stk1* strains as described [69]. Kaplan-Meier survival curves shown in Fig. 4A indicate that 100% of mice infected with WT *S. aureus* Newman succumbed to infection, whereas 90% of the mice infected with a Δ*stp1* mutant survived the infection (*P* value<0.0001, log rank test). Similar results was also observed with the Newman *stp1*::TN5EZ strain (data not shown). Although the mice infected with a Δ*stk1* mutant did not show a significant difference in survival compared to the WT (*P* value = 0.44), a large number of abscesses were observed in the kidneys of mice infected with this strain (Fig. 4B). In contrast, the kidneys obtained from mice infected with a Δ*stp1* mutant showed no visible abscesses (Fig. 4B). Histological examination of infected kidneys at five days post-inoculation indicated the presence of massive abscesses and necrotic tissue surrounding bacterial microcolonies in mice infected with WT or a Δ*stk1* mutant, but not in those infected with a Δ*stp1* mutant (Fig. 4C). These data emphasize the importance of Stp1 and Stk1 for *S. aureus* virulence and abscess formation.

**Figure 5:**
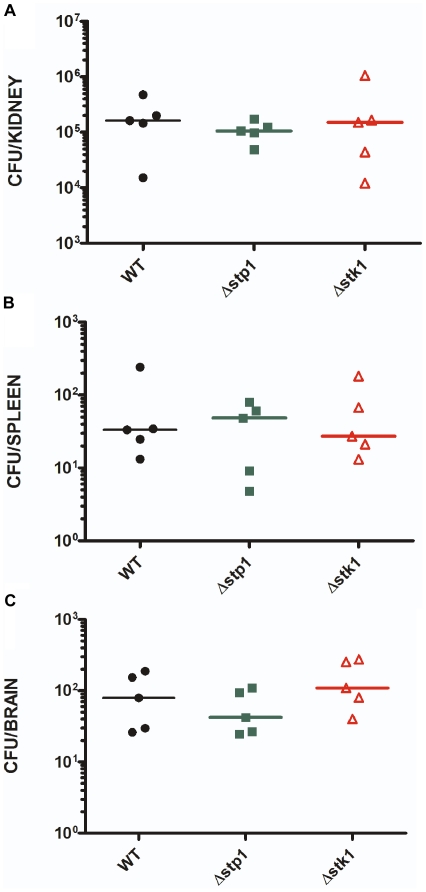
Stp1 and Stk1 do not regulate *in vivo* proliferation of *S. aureus*. Five, six week old female C57Black6 mice were intravenously injected with 1×10^7^ CFU of WT, Δ*stp1* or Δ*stk1* strains. At approximately 5 days post-infection, kidneys, spleen, and brains were harvested from infected mice and bacterial CFU enumerated. Note that the CFU of WT, Δ*stp1* or Δ*stk1 S. aureus* were not significantly different in the kidneys (**A**), spleens (**B**) or brains (**C**) of infected mice.

**Figure 6:**
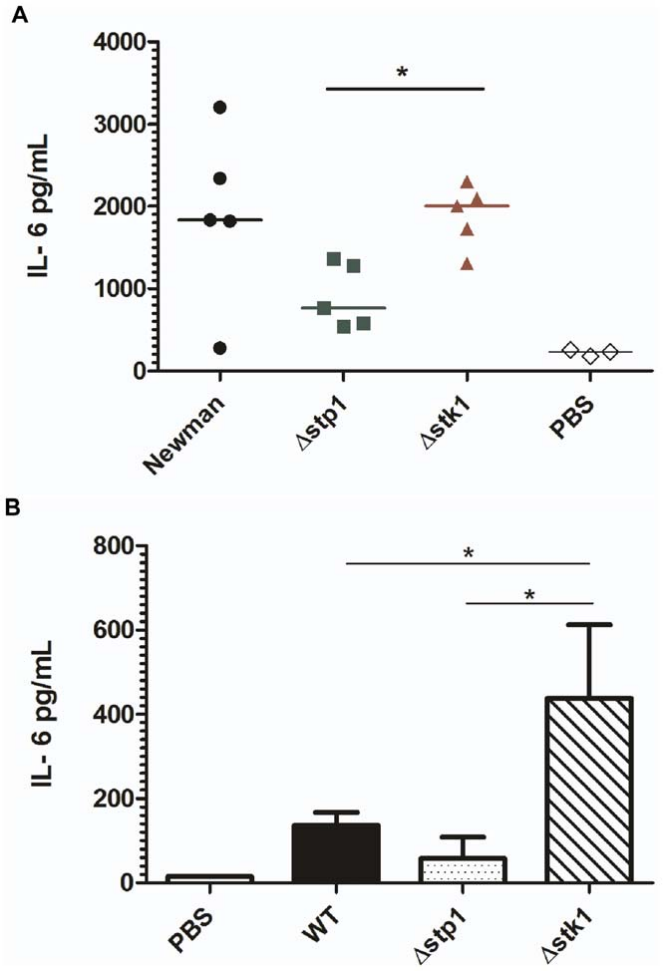
Stp1 and Stk1 regulate the host immune response to *S. aureus* Newman. (**A**) Cytokine IL-6 levels were measured in kidney homogenates obtained from infected mice at 5 days post-inoculation. IL-6 levels were significantly lower in kidneys obtained from Δ*stp1* mutant-infected mice compared to Δ*stk1* mutant-infected mice (*P* value = 0.003). (**B**) Bone marrow derived macrophages (BMMΦ) were stimulated with WT, Δ*stp1* or Δ*stk1 S. aureus* cells and cytokine IL-6 levels measured as described in the Methods. Secretion of IL-6 was increased in response to *S. aureus* Δ*stk1* in comparison to Δ*stp1* or WT (*P* value = 0.04). The experiment was repeated three times and similar results obtained. Data shown represent the mean and SD of one experiment.

The differences in survival and renal abscesses observed in the mice infected with the Δ*stp1* and Δ*stk1* strains prompted us to compare bacterial spread and cytokine responses during infection. Six week-old female C57BL/6J mice (*n* = 5) were inoculated intravenously with 1×10^7^ colony-forming units (CFU), and kidneys, spleen and brain were harvested at 5 days post-infection as described [69]. We observed that the bacterial CFU in the kidneys, spleen and brain of mice infected with the WT, Δ*stp1* or Δ*stk1* strains were not significantly different at 5 days post-infection (Fig. 5, A–C). These results indicate that the attenuated virulence of Δ*stp1* is not due to decreased survival or dissemination within the host. A comparison of the host inflammatory response to infection revealed a two-fold decrease in IL-6 production in the kidneys of mice infected with the Δ*stp1* strain compared to kidneys of mice infected with the Δ*stk1* strain (Fig. 6A). We did not observe significant changes in KC (the murine functional homologue of IL-8) and IL-1β production. A two-fold decrease in IL-6 expression was also observed when bone marrow-derived macrophages (BMMΦ) of C57BL/6 mice were stimulated with the Δ*stp1* mutant, compared to the Δ*stk1* strain (Fig. 6B). These studies indicate that the staphylococcal serine/threonine kinase and phosphatase affect the IL-6 response to *S. aureus* infection.

### Phosphopeptide enrichment analysis

**Figure 7:**
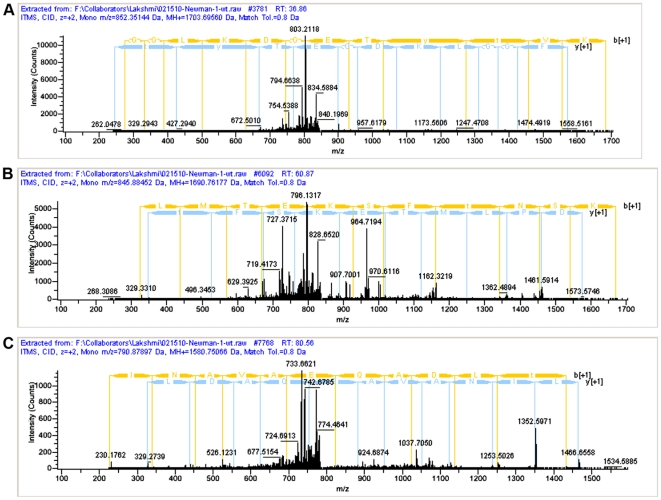
MS spectra of threonine phosphorylated peptides corresponding to SdrE (A), NWNM_1123 (B) and DNA binding protein HU (C). The mass spectra show neutral loss of phosphoric acid from phosphopeptides that were unique to WT Newman (*i.e.* not detected in the Δ*stk1* mutant). (**A**) the *m/z* of the doubly charged precursor ion corresponding to SdrE is 852.35144. Note that the peak depicting neutral loss of phosphoric acid (H_3_PO_4_) has an *m/z* of 803.2118 due to the loss of 49 Da from the doubly charged peptide. (**B**) Likewise, the peak depicting neutral loss of phosphoric acid (H_3_PO_4_) from the precursor ion corresponding to NWNM_1123 has an *m/z* of 796.1317 due to the loss of 49 Da from the doubly charged peptide. (**C**) Note that the peak depicting neutral loss of phosphoric acid (H_3_PO_4_) has an *m/z* of 742.6785 due to the loss of 49 Da from the doubly charged peptide corresponding to DNA binding protein HU.

To identify potential substrates of Stk1, we performed *in vivo* phosphopeptide enrichment and mass spectrometric analysis on total proteins isolated from WT Newman and the isogenic Δ*stk1* strain as described previously [70] with a few modifications (see Methods). Phosphopeptides (i.e. peptides that showed neutral loss of phosphate during collision induced dissociation, CID) corresponding to Stk1, DNA binding histone like protein (HU), serine-aspartate rich fibrinogen/bone sialoprotein binding protein (SdrE) and a hypothetical protein (NWMN_1123) were identified in the WT and not in the Δ*stk1* mutant (Table 6, Fig. 7A–C). These data suggest that Stk1 phosphorylates HU, SdrE and NWNM_1123 in *S. aureus*. As phosphopeptides corresponding to a NAD-specific glutamate dehydrogenase (GudB), pyruvate kinase (Pyk), anti-sigma B factor antagonist (RsbV), threonyl-tRNA synthetase (ThrS) and an acetyltransferase (NWNM_2273) were identified in both WT and the Δ*stk1* mutant (Table 6), phosphorylation of these proteins are likely to be independent of Stk1. Collectively, these studies suggest that post-translational regulation of HU, SdrE and NWNM_1123 by Stk1 and Stp1 affects *S. aureus* gene expression and virulence.

**Table 6:** *S. aureus* phosphopeptides identified using *in vivo* phosphopeptide enrichment and mass spectrometry.

Phosphopeptides unique to *S. aureus* WT Newman				
Locus	Phosphopeptide	Protein Description	Strain	
			WT	Δ*stk1*
NWMN_1123	PLMTEKSFT*NSK	Hypothetical protein	✓	×
HU	INAVAEQADLT*K	DNA-binding protein HU	✓	×
SdrE	GGLKDGETYT*VK	Ser-Asp rich fibrinogen/bone sialoprotein-binding protein,	✓	×
SdrE	TENNS*TNP	Ser-Asp rich fibrinogen/bone sialoprotein-binding protein	✓	×
Stk1	ALSETSLTQT*NHVLGTVQYFSPEQAK	Serine/threonine kinase	✓	×
**Phosphopeptides present in ** ***S. aureus*** ** WT Newman and the Δ** ***stk1*** ** mutant**				
GudB	DS*FGTVTNLFEETISNK	NAD-specific glutamate dehydrogenase	✓	✓
Pyk	ALGLITEENGIT*SPSAIVGLEK	Pyruvate kinase	✓	✓
RsbV	MDS*TGLGLFVGTLK	Anti-sigma B factor antagonist	✓	✓
ThrS	IGKELELFTNS*QLV	Threonyl-tRNA synthetase	✓	✓
NWMN_2273	MVKVT*YDIPTCEDY	Acetyltransferase, GNAT family protein	✓	✓

# Peptides listed above showed neutral loss of phosphoric acid in the LC/MS analysis. The serine or threonine residue that is phosphorylated is indicated as S* or T*, respectively. A ‘✓’ indicates that the phosphopeptide was identified in the mass spectrometric analysis from the corresponding strain. A ‘×’ indicates that the phosphopeptide was not identified in the mass spectrometric analysis from the corresponding strain. NWMN numbers correspond to the ORF of the gene in the genome sequence.

### Cell morphology, antibiotic resistance or growth in minimal media

Recently Beltramini *et al.* reported that an *stk1* mutant derivative of *S. aureus* N315 was more susceptible to cell wall-acting antibiotics and showed morphological changes such as incomplete septa and irregular cell size, whereas an *stp1* mutant had increased peptidoglycan (PG, [71]). In contrast to these findings, we observed no significant differences in the sensitivity of WT Newman, Δ*stk1* and Δ*stp1* strains to the cell wall-acting antibiotics imipenem, ceftriaxone and the fluoroquinolone ciprofloxacin (MIC = 0.047, 4 and 0.125 µg/mL, respectively). Furthermore, cross-sectional TEM did not reveal any irregularities in PG thickness or cell shape of these strains (Fig. S1).

Other studies have suggested that Stk1 phosphorylates PurA (adenylosuccinate synthase) in *S. aureus* strain 8325 to regulate purine biosynthesis [72]. To determine whether Stk1 and Stp1 are important for purine biosynthesis in *S. aureus* Newman, we compared the ability of WT, Δ*stk1* and Δ*stp1* strains to grow in chemically defined media (CDM) with or without exogenous purines and/or pyrimidines as described [73]. The Δ*stk1* and Δ*stp1* mutants did not exhibit any significant differences during growth in chemically defined media (CDM) supplemented with purines and pyrimidines, CDM supplemented only with either purines or pyrimidines, and CDM lacking both purines and pyrimidines (see Fig. S2). As expected, the control strain with the transposon Tn5EZ insertion in *purA* was unable to grow in the absence of exogenous purines (Fig. S2) and was also avirulent in the mouse sepsis model of infection (data not shown). The apparent differences in the roles of Stk1 and Stp1 may be due to differences in target substrates in *S. aureus* strains.

## Discussion

In recent years, invasive infections due to *S. aureus* have escalated in hospital and community settings. The importance of hemolysins such as α toxin to *S. aureus* virulence has been extensively characterized. Given that *S. aureus* commonly resides as a commensal in the nasal passages and gastrointestinal tract but is also capable of invading deeper tissues, the pathogen must appropriately express hemolysins and other virulence factors in response to its external environment. To identify novel regulators of the hemolysins, we screened a transposon insertion library for mutants that showed altered toxin expression. Our studies identified 72 genes that affect the hemolytic activity of *S. aureus*. While some of the identified genes include previously established regulators such as *agr*, *sae*, *rot* and *ccpA*, we also identified novel genes that repress or activate the expression of *S. aureus* hemolytic toxins. Notably, insertions in a putative cyclic di-GMP synthetase decreased hemolysin expression, whereas insertions in purine biosynthetic enzymes, a LysR family transcriptional regulator and a serine/threonine kinase enhanced hemolysin expression. Given that spontaneous mutations in the *agr* locus have been associated with changes in hemolysin expression in *S. aureus*, we confirmed that *agr* mutations are not responsible for the hemolysin phenotypes of the Nemwam Δ*stp1* and Δ*stk1* mutants. Although similar studies need to be performed with the other genes identified in this study, a few loci that were identified as regulators of toxin expression such as lipoamide dehydrogenase (SA1563), dihydrodipicolinate synthase (*dapA*) and phosphoribosylformylglycinamidine synthase (*purL*) were previously identified in screens for *S. aureus* mutants that showed virulence attenuation [74], [75], [76], [77].

Signal transduction in bacteria classically involves the reversible phosphorylation of histidine residues, whereas phosphorylation of serine/threonine residues was originally described in eukaryotes. However, examples of eukaryotic-type serine/threonine phosphorylation are increasingly encountered in bacteria. Previous studies from our laboratory have indicated that a serine/threonine kinase regulates hemolysin expression in Group B Streptococcus [60], [61]. Therefore, we further characterized the role of the kinase Stk1 and its cognate phosphatase Stp1 in the regulation of *S. aureus* hemolysin expression and virulence. As the *S. aureus* strain RN4220 used to construct the transposon library is not virulent, we evaluated the effect of transposon insertions in *stk1* and *stp1* in a virulent WT *S. aureus* strain. A comparison of hemolysin expression in the mutant strains revealed that Stk1 negatively affects transcription of α toxin whereas the phosphatase Stp1 promotes α toxin expression in virulent *S. aureus* Newman. The identification of a phosphopeptide corresponding to the DNA binding histone like protein (HU) suggests that *S. aureus* modulates the function of HU for regulation of gene expression similar to histone phosphorylation in eukaryotes [78], [79]. It has also been suggested that HU binding introduces structural changes to the DNA which can facilitate or inhibit the binding of regulatory proteins to their specific sites. Recently in *E. coli*, HU was described to regulate the expression of ∼8% of the genome [80]. As *hla* transcription is decreased in Δ*stp1* and increased in Δ*stk1*, we predict that HU may regulate *hla* transcription in *S. aureus*. Likewise, changes in gene expression observed in the Δ*stp1* and Δ*stk1* strains could also be attributed to altered HU function. In *S. aureus*, HU is encoded by the gene *hsa* and is essential for growth [81]. Further studies to identify all genes that are directly regulated by HU are being pursued and will reveal the role of post translational regulation of HU in *S. aureus*.

Given the importance of *S. aureus* adherence to extracellular matrix components, we hypothesize that the increase and decrease in kidney abscesses observed with the Δ*stk1* and Δ*stp1* strains may, in part, be linked to reversible phosphorylation of SdrE. SdrE is a member of the family of surface proteins with serine (S)–aspartate (D) repeats (R), and also includes SdrC, SdrD and clumping factors (ClfA and ClfB). These proteins belong to a sub-family of adhesins that specifically bind extracellular matrix molecules and are also known as microbial surface components recognizing adhesive matrix molecules (MSCRAMM, [82], [83]). Although an exact molecular function for SdrE is not known, it is proposed to bind host extracellular matrix components and has been shown to contribute to platelet aggregation [84], [85]. Immunization with the cell surface proteins SdrD and SdrE along with two other antigens generated significant protective immunity [84]. Furthermore, *S. aureus* with an *sdrE*-positive gene profile are associated with bone infections [83]. Further studies to understand the role of phosphorylation on SdrE function and *S. aureus* abscess formation and virulence are being pursued. Likewise, similar studies to understand the role of the hypothetical protein NWNM_1123 are also being investigated to establish the role of phosphorylation of the Stk1 targets in regulation of gene expression and *S. aureus* virulence.

In contrast to our findings, Beltramini *et al* recently concluded that Stp1 and Stk1 do not regulate hemolysin expression in the methicillin resistant *S. aureus* (MRSA) strain N315 [71]. However, the role of either Stp1 or Stk1 in the virulence of strain N315 was not examined [71]. Recently, *stk1* mutations generated in *S. aureus* strain SH1000 showed attenuated virulence which was attributed to purine auxotrophy [72], [86]. In contrast, we observed that Stp1 and Stk1 are dispensable for purine biosynthesis in *S. aureus* Newman (Fig S2), and a Δ*stk1* mutation does not attenuate virulence in this strain background (Fig. 4). Collectively, these observations suggest that Stk1 and Stp1 may mediate the phosphorylation of different targets in *S. aureus* strains Newman, N315 and SH1000. Identification of the *in vivo* Stk1 substrates in N315 and SH1000 will elucidate the role of serine/threonine phosphorylation in these *S. aureus* strains. Although the strain Newman has been extensively used to study *S. aureus* pathogenesis, recent studies have indicated the presence of a missense mutation in the sensor histidine kinase SaeS that affects downstream gene regulation [87], [88] Consequently, evaluation of the role of Stk1 and Stp1 in hemolysin expression and *S. aureus* virulence in clinical isolates such as those of the USA300 lineage, are in progress in our laboratory and, will provide further significance to our observations in *S. aureus* Newman.

In this study, we have also identified a number of novel genes encoding a putative LysR family transcription factor and a putative cyclic di-GMP synthetase that regulate hemolysin expression in *S. aureus*. Further characterization of these loci will establish novel mechanisms by which the expression of exotoxins and other *S. aureus* virulence genes are regulated.

## Methods

### Ethics Statement

All animal experiments were approved by the Institutional Animal Care and Use Committee of Seattle Childrens Research Institute (IACUC protocol # 08-0515) and performed using accepted veterinary standards.

### Bacterial strains and growth conditions

A *S. aureus* RN4220 random transposon library containing 6,725 individual Tn5EZ mutants and the location of the these insertions were kindly provided by Novartis Vaccines and Diagnostics (CMCC #51963, see patent WO/2004/018624 entitled ‘Random transposon insertion in *Staphylococcus aureus* and use thereof to identify essential genes’ at http://www.wipo.int/pctdb/en/wo.jsp?WO=2004%2F018624&IA=WO2004%2F018624&DISPLAY=DESC). All other strains, plasmids and primers used in this study are listed in Table S1. *S. aureus* strains were cultured in Tryptic Soy Broth (Difco Laboratories, Detroit, MI) at 37°C unless indicated otherwise. The WT *S. aureus* strains used in this study are RN4220 and Newman. Newman is a clinical isolate of *S. aureus* that belongs to the capsular polysaccharide serotype 5 and is routinely used in virulence studies [8], [21], [69], [89], [90]. Routine cultures of *E. coli* were performed in Luria-Bertani broth (Difco Laboratories, Detroit, MI) at 37°C. Cell growth was monitored by reading optical density at 600 nm. Antibiotics were added at the following concentrations when necessary: for *E.coli*, ampicillin 100 µg/mL, kanamycin 50 µg/mL, erythromycin 300 µg/mL, chloramphenicol 10 µg/mL; for *S. aureus*, kanamycin 50–100 µg/mL, erythromycin 1–5 µg/mL and chloramphenicol 10 µg/mL. All chemicals were purchased from Sigma-Aldrich (St. Louis, MO), and molecular biology reagents were purchased from New England Biolabs or Promega Corporation, USA.

### Construction of *S. aureus* allelic exchange mutants

Approximately 1kb of DNA flanking the 5′ and 3′ ends of *stp1* or *stk1* were PCR-amplified from *S. aureus* Newman using High fidelity PCR (Invitrogen CA, USA). The primer pairs used to amplify 1kb regions located upstream and downstream of *stp1* were SAStp1000upF and SaSTP1000upCatR, and SaSTKcodingCatF and SAStkcodingR2, respectively. Likewise, the primer pairs used to amplify 1kb regions located upstream and downstream of *stk1* were PSAF3 and SAstpnewKanR, and PSAF2+ and PSAR4, repectively. For allelic replacement of *stp1*, the gene conferring chloramphenicol (*cm*) resistance was amplified from the plasmid pDC123 [64] using the primers SA-CatF and SA-CatR. For alleic replacement of *stk1*, the gene conferring kanamycin (*kn*) resistance was amplified from pCIV2 [65] using the primers SA-KanF and SA-KanR. Subsequently, SOEing PCR [91] was performed to introduce the antibiotic resistance gene (*cm* or *kn*) between the flanking regions of *stp1* or *stk1*. The PCR fragments were then ligated into the temperature sensitive vector pHY304, [92]) and the resulting plasmids pKB8 (Δ*stp1::cm*) and pKB1 (Δ*stk1::kn*) were electroporated into *S. aureus* strain RN4220 using methods described previously [69]. Selection for single crossovers and screening for the double crossovers was performed as described [60], [69]. PCR was used to verify the presence of the allelic replacement cassette and the absence of the gene of interest. Subsequently, the above mutations were transduced into the virulent *S. aureus* wild type (WT) strain Newman using phage phi11 (φ11) as described [62]. PCR was used to verify the presence of the allelic replacement cassette in the region of interest in *S. aureus* Newman. Complemented strains were constructed using the vector pDCerm [93]. The genes encoding *stp1* and *stk1* were amplified using the primers SaStpBamHIR and SaStpEcoRIF for *stp1*, and pDCSaStkBamHIR and pDCSaStkEcoRIF for *stk1*, and ligated into pDCerm to generate pStp1 and pStk1, respectively (Table S1).

### Quantitative western blots

*S. aureus* WT Newman and isogenic mutants were grown in TSB to post-exponential phase (OD_600_∼3.0). The cells were centrifuged and secreted proteins were precipitated from cell-free supernatants using 25% TCA as described previously [21]. Cell pellets were disrupted using the FastPrep FP101 bead beater (Bio 101) and centrifuged to remove unlysed cells and cell debris. Intracellular or cytoplasmic proteins were quantified in the supernatants using the Bradford protein assay [94]. Equal amounts of secreted or cytoplasmic proteins (40µg) from each strain were subjected to 4–12% SDS-PAGE (Invitrogen NuPAGE). One µg of purified α toxin (Sigma Chemical Co., USA) was included as a positive control. Following electrophoresis, the proteins were transferred to a PVDF membrane as described [94]. The membrane was blocked in Odyssey buffer (Licor, USA) containing 5% normal goat serum. Subsequently, α toxin antibody (Sigma Chemical Co., USA) was added at a 1∶10,000 dilution followed by secondary Alexa Fluor goat anti-rabbit antibody (Invitrogen) at 1∶10,000. Washes and imaging were performed as per manufacturer's instructions for the Odyssey Licor infrared imager.

### Isolation and purification of total RNA

Total RNA was isolated using the RNeasy Mini kit (QIAGEN, Inc., Valencia, CA). In brief, *S. aureus* strains were grown to an OD_600_ of 0.6 or ∼3.0, centrifuged, and washed in 1∶1 mixed RNA Protect and TE buffer (QIAGEN, Inc., Valencia, CA). The cells were then resuspended in RLT buffer (QIAGEN, Inc., Valencia, CA) and lysed using a FastPrep FP101 bead beater (Bio 101) with 3×30 s bursts at a power setting of 6, followed by centrifugation. RNA was isolated from the supernatants using the RNeasy Mini kit as described by the manufacturer (QIAGEN, Inc., Valencia, CA). RNA concentration and integrity was determined using an Agilent 2100 Bioanalyzer (Agilent, Santa Clara, CA) and a NanoDrop 1000 (NanoDrop, Wilmington, DE) for subsequent use in qRT-PCR and microarray.

### Quantitative Real-Time Reverse Transcription PCR (qRT-PCR)

RNA isolated from three independent biological replicates for each strain was purified as described above. qRT-PCR was performed using a two-step QuantiTect SYBR Green RT-PCR kit (QIAGEN, Inc., Valencia, CA) following the manufacturer's instructions for a total reaction volume of 20 µL (iCycler thermocycler; Bio-Rad, Hercules, CA). cDNA synthesis was achieved using 50 ng of total RNA, and PCR amplification was performed as recommended by the manufacturer (Qiagen, USA). All runs were immediately followed by a melting curve analysis to evaluate PCR specificity and showed single primer-specific melting temperatures. All assays were performed with at least three independent biological replicates and three technical replicates for each gene. All primers were calculated using Primer3 services (http://frodo.wi.mit.ed/cgi-bin/primer3_www.cgi), purchased from Integrated DNA Technologies (Coralville, IA) or Sigma Chemical Company, and are listed in Table S1. The housekeeping gene *rpoD* was used to normalize transcript measurements, and relative gene expression calculated using the comparative C_T_ method [68]. Statistical significance was determined students t test as described previously [61].

### Microarray

For microarray analysis, RNA was isolated from two independent biological replicates of each strain as described above. Purified RNA was sent to NimbleGen Systems, Inc. (Madison, WI) for full expression services. NimbleGen performed: cDNA synthesis, labeling of the cDNA, and hybridization of the labeled cDNA to the *Staphylococcus aureus COL* custom chip (090327_Saur_COL_SACOL1232_Expr_X4; NimbleGen Systems, Inc. Madison, WI) as per company protocols. The chips were composed of 9 probes per target sequence, and each probe was replicated three times (http://www.nimblegen.com/products/exp/prokaryotic.html). Microarray data was interpreted, analyzed and statistically evaluated using the program GeneSpring GX (GeneSpring GX 7.3.1; Agilent Technologies, Santa Clara, CA). All fold changes were defined relative to the Newman WT strain. All data is MIAME compliant and that the raw data has been deposited in a MIAME compliant database (GEO, accession number GSE21426), as detailed on the MGED Society website http://www.mged.org/Workgroups/MIAME/miame.html.

### Virulence Assay

All animal experiments were approved by the Institutional Animal Care and Use Committee of Seattle Childrens Research Institute and performed using accepted veterinary standards. The *S. aureus* WT strain Newman and isogenic mutants were used for analysis of virulence as described previously [69], [89], [90]. Briefly, approximately 2–5×10^7^ CFU of each *S. aureus* strain was injected via the tail vein into 6 week-old female C57BL/6J mice (*n* = 10) purchased from Jackson Laboratories (Bar Harbor, ME). Infected mice were monitored every 12 hrs for signs of morbidity. Kidneys were obtained from mice that were moribund or at the end of an experiment, visually examined for abscess formation, and homogenized for CFU enumeration. To compare CFU between WT, Δ*stp1* and Δ*stk1* strains, 1×10^7^ CFU of each *S. aureus* strain was injected via the tail vein into 6 week-old female C57BL/6J mice (*n* = 10), and spleen, kidneys and brain were harvested from infected mice (*n* = 10 per group) 5 days after infection. Bacterial CFU were enumerated from the homogenized tissue of five mice by plating serial 10-fold dilutions on TSB agar. Kidneys from the remaining mice (*n* = 5) were fixed in formalin, embedded in paraffin, thin-sectioned, stained with hematoxylin and eosin (H&E) and examined by microscopy as described previously [69].

### Host immune response

IL-6 ELISA assays were performed on kidney homogenates from above using the DuoSet® kit as described by the manufacturer (R&D Systems, USA). Bone marrow derived macrophages (BMMΦ) were derived as described previously [95] with some modifications. Briefly, for each experiment, femurs were excised from three C57BL/6J mice and bone marrow cells were flushed, washed and resuspended using complete medium (RPMI+20% fetal calf serum+l-Glutamine (2mM)). Two million bone marrow cells were then differentiated into macrophages using 20 ml complete medium supplemented with murine 40 ng/ml rM-CSF (PeproTech). Cells were harvested between days 7–10, at which point >99% of the cells were F4/80 Antigen^+^. BMMΦ were transferred to 96-well plates (7×10^4^ cells in 200 µl medium/well) and stimulated with WT, Δ*stp1* or Δ*stk1* cells for 24h at MOI of 1. Supernatants were collected and stored at −80°C for ELISA assays. IL-6 ELISA assays were performed using the DuoSet® kit as described by the manufacturer (R&D Systems, USA). All experiments were repeated three times in triplicate.

### Statistical analysis

The Mann-Whitney test was used to evaluate differences between cytokine levels and CFU/organ between *S. aureus* strains. Survival analyses were performed using a log rank test. These tests were performed using GraphPad Prism version 5.00 for Windows, GraphPad Software, San Diego California USA, www.graphpad.com.

### Phosphopeptide enrichment and Mass spectrometry

Total protein was isolated from WT Newman and Δ*stk1* mutant as described previously [70] with a few modifications. Briefly, each sample was normalized to contain equal amount of protein and the proteins were denatured and reduced in 10mM DTT containing 0.1% Rapigest (Waters, USA) at 50°C for 30 min. Subsequently, the reduced cysteines were alkylated with 30mM iodoacetamide for 1hr in the dark and the samples were digested overnight at 37°C using sequencing grade trypsin (1∶100,trypsin∶total protein). Rapigest was removed and samples were desalted using Sep-Pak C-18 columns according to manufacturer's instructions (Waters, USA) and dried using a Speedvac. From the peptide samples of each strain (500µg), phosphopeptides were enriched and captured using soluble nanopolymer (PolyMAC) as described previously [96]. Unbound non-phosphopeptides were washed and phosphopeptides were eluted as described previously [96]. The samples were analyzed by capillary liquid chromatography- nanoelectrospray tandem mass spectrometry (μLC-nanoESI-MS/MS) using a high resolution hybrid linear ion trap orbitrap (LTQ-Orbitrap Velos, Thermo Fisher) coupled with Eksigent Ultra2D nanoflow HPLC and methods described previously [70], [96]. Data were searched using Proteome Discoverer™ software with Sequest™ algorithm at 10 ppm precursor mass accuracy cuttoff. The searches included static modification on Cys residues (+57.0214), and variable modifications on methionine (+15.9949) and Ser and Thr residues (+79.997) to identify phosphorylation as described previously [70], [96]. Spectra were searched against the *S. aureus* strain Newman database (NC_009641) with a 5% FDR cutoff based on the reverse database decoy search using methods described [96]. The experiment was performed using two independent biological replicates of each strain.

## Supporting Information

Figure S1 Cell morphology of stp1 and stk1 mutants is similar to the WT *S. aureus* Newman. Cross sectional transmission electron micrographs shown are at a magnification of 100,000. Arrows show regions of PG that are marked with a line of the same size across the three panels. Significant differences in cell morphology or thickness of peptidoglycan are not apparent between WT Newman and isogenic stp1 and stk1 mutants.
(0.91 MB DOC)

Figure S2 Stp1 and Stk1 are dispensable for purine and pyrimidine biosynthesis of S. aureus Newman. *S. aureus* strains were grown in chemically defined media (CDM) at 37°C and cell growth was monitored every half hour. The OD600 of the strains after 5 hrs of growth is shown. CDM was prepared as described (Richardson et al., 2006, Molecular Microbiology 61: 927–939). ‘Complete CDM (cCDM)’ represents CDM containing exogenous purines (adenine, guanine and xanthine) and the pyrimidine (uracil). ‘Deleted CDM (dCDM)’ represents CDM lacking both purines and pyrimidines. ‘dCDM + Purines’ represents CDM that had the purines (adenine, guanine and xanthine) but did not contain pyrimidines. ‘dCDM + Pyrimidines’ represents CDM that was supplemented only with uracil and lacked purines. Note that the control strain with a transposon insertion in purA showed no growth in media lacking purines. Growth and doubling time of the stp1 and stk1 mutants was comparable to WT S. aureus Newman.
(0.03 MB DOC)

Table S1 Strains, Plasmids, and Primers.
(0.07 MB DOC)

Table S2 Genes with altered expression in stp1 and stk1 mutants at post-exponential phase.
(0.25 MB DOC)
