# Supplementary figures and images for: Regulation of Hemolysin Expression and Virulence of Staphylococcus aureus by a Serine/Threonine Kinase and Phosphatase
Source: PLoS One. 2010 Jun 11;5(6):e11071. doi: 10.1371/journal.pone.0011071 (PMC2884019; doi:10.1371/journal.pone.0011071)

**
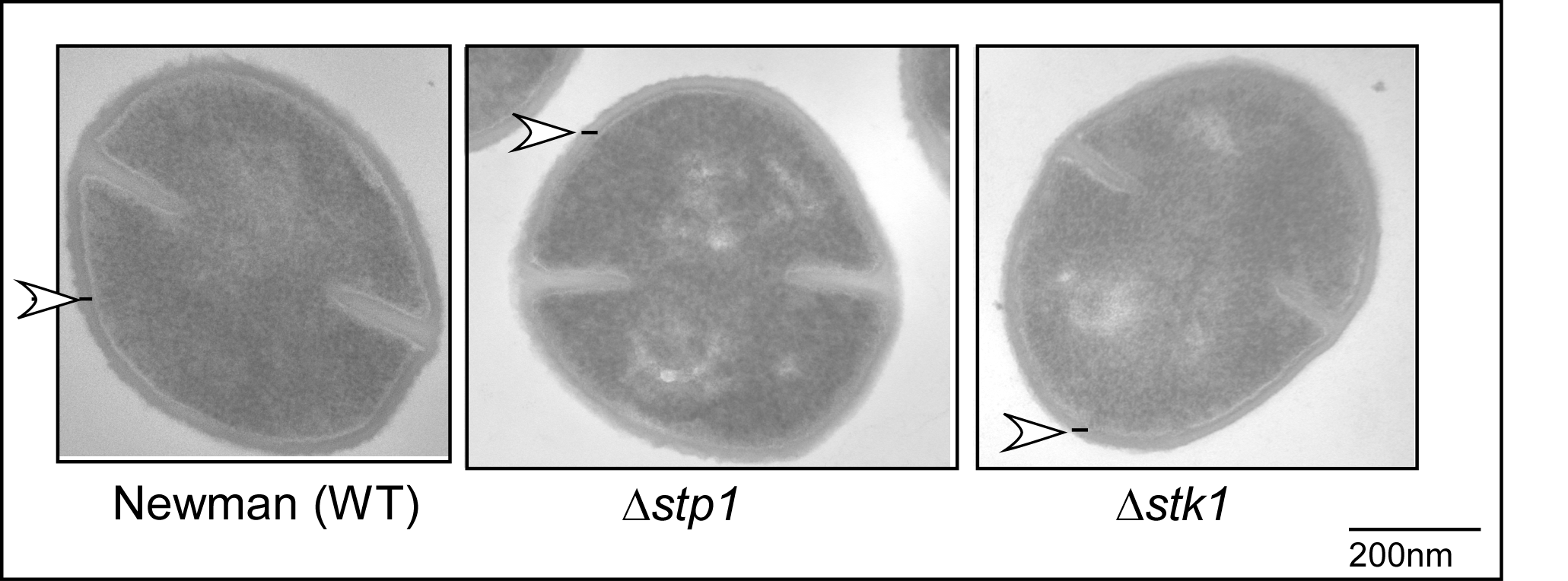
**

Supplement: Figure S1 — Cell morphology of stp1 and stk1 mutants is similar to the WT S. aureus Newman. Cross sectional transmission electron micrographs shown are at a magnification of 100,000. Arrows show regions of PG that are marked with a line of the same size across the three panels. Significant differences in cell morphology or thickness of peptidoglycan are not apparent between WT Newman and isogenic stp1 and stk1 mutants. (0.91 MB DOC) [file pone.0011071.s001.doc]
