# Supplementary material for: Regulation of Hemolysin Expression and Virulence of Staphylococcus aureus by a Serine/Threonine Kinase and Phosphatase
Source: PLoS One. 2010 Jun 11;5(6):e11071. doi: 10.1371/journal.pone.0011071 (PMC2884019; doi:10.1371/journal.pone.0011071)
Supplement: Table S1 — Strains, Plasmids, and Primers. (0.07 MB DOC) [file pone.0011071.s003.doc]

| **Strains** | **Genotype\phenotype** | **Reference** |
| --- | --- | --- |
| ***Staphylococcus aureus*** | | |
| RN4220 | MNNG mutagenized strain of 8325-4 that accepts foreign DNA | Kreiswirth *et al*, 1983 |
| Newman | Wild type (WT), human clinical isolate | Duthie  1952 |
| *Δstk1* | Newman Δ*stk1*::*Ωkm-2*, Kn^R^ | This study |
| *Δstp1* | Newman Δ*stp1*::*ΩCm*, Cm^R^ | This study |
| *Δstk1/*pStk1 | Newman Δ*stk1*::*Ωkm-2*, Kn^R^*/*pStk1 | This study |
| *Δstp1/*pStp1 | Newman Δ*stp1*::*ΩCm*, Cm^R^*/*pStp1 | This study |
| ***Escherichia coli*** | | |
| MC1061 | F’*araD139 Δ(ara-leu)7696 Δ(lac)X74 galU galK hadR2* (r_k_^-^ m_k_^+^) *mcrB1 rpsL* (Str^+^) | Wertman *et al.*  1986 |
| DH5α | *fhuA2* Δ*(argF-lacZ)U169 phoA glnV44 Φ80* Δ*(lacZ)M15 gyrA96 recA1 relA1 endA1 thi-1 hsdR17* | Bethesda Research Laboratories, 1986 |
| **Plasmids** | | |
| pHY304 | Em^R^, temperature sensitive shuttle vector | Chaffin *et al.* 2000 |
| pKB01 | pHY304 encoding Δ*stk1*::*Ωkm-2* insert | This study |
| pKB08 | pHY304 encoding Δ*stp1*::*ΩCm* insert | This study |
| pStp1 | pDCerm encoding *stp1* | This study |
| pStk1 | pDCerm encoding *stk1* | This study |
| pDCerm | Complementation vector, Em^R^ | Jeng *et al.* 2003 |

| **Primers** | | |
| --- | --- | --- |
| **Primer name** | | **Sequence** |
| SAStp1000upF | | 5’-GTCTTCTAGAAAAACTGGCTCGTTGAACAAG-3’ |
| SaSTP1000upCatR | | 5'TAAAGTCAATATTACTGTAACATAATTGTCTTTACCTCGTTT  CTAC -3' |
| SaSTKcodingCatF | | 5'- GACCTAATGACTGGCTTTTAATTGAAGGTGATAAAGTAT  G-3' |
| SAStkcodingR2 | | 5’-TAATCTCGAGTTGCTGATGATGAGCAGG-3’ |
| PSAF3 | | 5’GCTGACTGAATTCACGCTTGATGCCAATTAACCGTGCATA  TAATGTTG-3’ |
| SAstpnewkanR | | 5’CCAATTCACTGTTCCTTGCATCATACTTTATCACCTTCA-3’ |
| PSAF2+ | | 5’-TTTTCTGAAGTACATCCGCACCGAGGTTTCTATTTGGAA  GTC-3’ |
| PSAR4 | | 5’-GCTGACCTCGAGCAAAATCTAAAGCACTGAATCCAGGTG  TGTCTGCAA-3’ |
| Sa-KanF | | 5'- TGCAAGGAACAGTGAATTGG-3' |
| Sa-KanR | | 5'- TGCGGATGTACTTCAGAAAAG-3' |
| SA-CatR | | 5’-TAAAAGCCAGTCATTAGGTC-3’ |
| SA-CatF | | 5’-TTATGTTACAGTAATATTGACTTTA-3’ |
| SaStpBamHIR | | 5'AGTCGGATCCTCATACTTTATCACCTTCAATAGC-3' |
| SaStpEcoRIF | | 5'-TACAGAATTCATGCTAGAGGCACAATTTTTTAC-3' |
| pDCSaStkBamHIR | | 5'-TGAGGGATCCTTATACATCATCATAGCTGACTTC-3' |
| pDCSaStkEcoRIF | | 5'-AATCGAATTCATGATAGGTAAAATAATAAATG-3' |
| **qRT-PCR Primers** | | |
| **Gene** | **Primer name** | **Sequence** |
| *hla* | hla-qRT-F | 5'-GCAAATGTTTCGATTGGTCA-3' |
|  | hla-qRT-R | 5'-CCATATACCGGGTTCCAAGA-3' |
| *hld* | hld-qRT-F | 5'- TAATTAAGGAAGGAGTGATTTCAATG -3' |
|  | hld-qRT-R | 5'- TTTTTAGTGAATTTGTTCACTGTGTC -3' |
| *agrA* | agrA-qRT-F | 5'- CGAAGACGATCCAAAACAAAG-3' |
|  | agrA-qRT-R | 5'- ATGTTACCAACTGGGTCATGC-3' |
| *spa* | spa-qRT-F | 5'- CAAACGGCACTACTGCTGAC-3' |
|  | spa-qRT-R | 5'- CATGGTTTGCTGGTTGCTTC-3' |
| *rpoD* | rpoD-qRT-F | 5’-GGAATAACATACCACGACCTA-3’ |
|  | rpoD-qRT-R | 5’-AAATGACCCAGTTCGTATGTACC-3’ |

Em^R^ erythromycin resistant, Cm^R^ chloramphenicol resistant, Kn^R^ kanamycin resistant
