# Supplementary material for: Regulation of Hemolysin Expression and Virulence of Staphylococcus aureus by a Serine/Threonine Kinase and Phosphatase
Source: PLoS One. 2010 Jun 11;5(6):e11071. doi: 10.1371/journal.pone.0011071 (PMC2884019; doi:10.1371/journal.pone.0011071)
Supplement: Table S2 — Genes with altered expression in stp1 and stk1 mutants at post-exponential phase. (0.25 MB DOC) [file pone.0011071.s004.doc]

| **Locus** | **Protein description or name** | **Δ*stk1*** | **Δ*stp1*** |
| --- | --- | --- | --- |
| **Pathogenesis** | |  |  |
| SACOL0442 | staphylococcal enterotoxin, super antigen like proteins | 4.88 | 2.29 |
| SACOL0474 | exotoxin 4, putative | 3.13 | 2.15 |
| SACOL0468 | exotoxin 3, putative | 3.06 | NS |
| SACOL2004 | leukocidin subunit precursor, LukF | 2.83 | NS |
| SACOL0472 | exotoxin, putative | 2.62 | 2.02 |
| SACOL0473 | exotoxin 5, putative | 2.59 | NS |
| SACOL2006 | Aerolysin/Leukocidin family protein, LukS | 2.14 | NS |
| SACOL0469 | exotoxin 1, putative | 2.07 | NS |
| SACOL0470 | exotoxin, putative | 1.97 | NS |
| SACOL1173 | alpha-hemolysin precursor (Hla, α toxin) | 1.8 | 0.6 |
| SACOL0142 | UDP-N-acetylglucosamine 2-epimerase Cap5G | 0.61 | 2.90 |
| SACOL0144 | capsular polysaccharide biosynthesis protein Cap5I | 0.61 | 2.72 |
| SACOL0148 | capsular polysaccharide biosynthesis galactosyltransferase Cap5M | 0.61 | 2.25 |
| SACOL0145 | capsular polysaccharide biosynthesis protein Cap5J | 0.60 | 2.79 |
| SACOL0146 | capsular polysaccharide biosynthesis protein Cap5K | 0.59 | 2.79 |
| SACOL0149 | capsular polysaccharide biosynthesis protein Cap5N | 0.57 | 2.21 |
| SACOL0147 | capsular polysaccharide biosynthesis protein Cap5L | 0.55 | 2.13 |
| SACOL0143 | capsular polysaccharide biosynthesis protein Cap5H | 0.54 | 2.91 |
| SACOL0141 | capsular polysaccharide biosynthesis protein Cap5F | 0.52 | 2.35 |
| SACOL0140 | capsular polysaccharide biosynthesis protein Cap5E | 0.51 | 2.28 |
| SACOL0139 | capsular polysaccharide biosynthesis protein Cap5D | 0.49 | 2.02 |
| SACOL0138 | capsular polysaccharide biosynthesis protein Cap5C | 0.48 | 2.29 |
| SACOL0136 | capsular polysaccharide biosynthesis protein Cap5A | 0.48 | 2.13 |
| SACOL0137 | capsular polysaccharide biosynthesis protein Cap5B | 0.48 | 2.07 |
| SACOL2509 | fibronectin binding protein B | 0.37 | 0.24 |
| SACOL2295 | staphyloxanthin biosynthesis protein, putative | 0.32 | NS |
| SACOL2584 | immunodominant antigen A | 0.24 | 0.4 |
| SACOL2581 | staphyloxanthin biosynthesis protein, SsaA homolog | 0.10 | 0.27 |
| SACOL2291 | staphyloxanthin biosynthesis protein, SsaA | NS | 0.49 |
| SACOL2511 | fibronectin-binding protein A | NS | 0.46 |
| SACOL0478 | exotoxin 3, putative | NS | 0.31 |
| **Signal Transduction/Transcriptional regulators** | |  |  |
| SACOL1942 | DNA-binding response regulator VraR | 2.39 | NS |
| SACOL1943 | sensor histidine kinase VraS | 2.23 | NS |
| SACOL2353 | transcriptional regulator TcaR | 2.11 | NS |
| SACOL2378 | transcriptional regulator, AraC family | 0.41 | 0.48 |
| SACOL0403^#^ | transcriptional antiterminator, BglG family | 0.34 | NS |
| SACOL0757 | transcriptional regulator, DeoR family | 0.31 | 0.14 |
| SACOL2585 | regulatory protein, putative | 0.27 | NS |
| SACOL1232 | Serine/threonine protein kinase Stk1 | 0.005 | NS |
| SACOL2732 | transcriptional regulator, putative | NS | 3.94 |
| SACOL0404 | transcriptional regulator, MarR family | NS | 0.35 |
| SACOL1904 | transcriptional regulator, putative | NS | 0.29 |
| SACOL0249 | transcriptional regulator, GntR family | NS | 0.25 |
| SACOL1231 | Serine/threonine protein phosphatase Stp1 | NS | 0.01 |
| **Protein secretion and Transporters** | |  |  |
| SACOL0086 | drug transporter, putative | NS | 3.09 |
| SACOL2514 | gluconate transporter, permease protein | NS | 2.74 |
| SACOL0685 | Na+/H+ antiporter, MnhF component, putative | NS | 2.29 |
| SACOL2708 | ABC transporter, ATP-binding protein | NS | 2.24 |
| SACOL1033 | ABC transporter, ATP-binding protein | NS | 2.24 |
| SACOL0422 | Na+/H+ antiporter, MnhE component, putative | NS | 2.22 |
| SACOL0491 | cobalt transport family protein | NS | 2.09 |
| SACOL2707 | Cobalt transport family protein | NS | 2.08 |
| SACOL2413 | drug resistance transporter, EmrB/QacA subfamily | NS | 2.05 |
| SACOL0302 | Branched-chain amino acid transport system II carrier protein | NS | 0.46 |
| SACOL2347 | drug resistance transporter, EmrB/QacA subfamily | NS | 0.44 |
| SACOL2279 | transporter, putative | NS | 0.37 |
| SACOL0501 | sodium-dependent transporter, putative | NS | 0.33 |
| SACOL0405 | MATE efflux family protein | NS | 0.33 |
| SACOL1897 | protein export protein PrsA, putative | 3.18 | NS |
| SACOL0159 | ABC transporter, permease protein | 2.61 | NS |
| SACOL0623 | hypothetical protein | 2.58 | NS |
| SACOL1475 | drug transporter, putative | 2.17 | NS |
| SACOL1476 | amino acid permease | 2.15 | NS |
| SACOL0417 | mttB family protein | 0.55 | 0.46 |
| SACOL2031 | ammonium transporter family protein | 0.47 | NS |
| SACOL0418 | mttA/Hcf106 family protein | 0.46 | 0.44 |
| SACOL2146 | PTS system, mannitol-specific IIBC components | 0.44 | 0.36 |
| SACOL0402 | PTS system, IIA component | 0.40 | NS |
| SACOL0250 | PTS system, IIA component | 0.39 | 0.26 |
| SACOL2619 | amino acid permease | 0.27 | NS |
| **Protein/peptide/amino acid and DNA synthesis/degradation** | | | |
| SACOL0085 | peptidase, M20/M25/M40 family | NS | 3.78 |
| SACOL2017 | chaperonin, 10 kDa | NS | 2.56 |
| SACOL1057 | V8 Protease | NS | 2.16 |
| SACOL1056 | cysteine protease precursor SspB | NS | 2.04 |
| SACOL1691 | Single stranded DNA specific exonuclease RecJ | NS | 0.48 |
| SACOL1869 | serine protease SplA | NS | 0.46 |
| SACOL0556 | chaperonin, 33 kDa | NS | 0.45 |
| SACOL1036 | protease, putative | NS | 0.43 |
| SACOL2072** | ATP dependent RNA helicase | NS | 0.33 |
| **Metabolic functions** | | | |
| SACOL1217 | orotate phosphoribosyltransferase | 2.65 | 3.35 |
| SACOL1932 | transglycosylase domain protein | 2.36 | NS |
| SACOL2515 | gluconokinase | 0.48 | 3.06 |
| SACOL1328 | glutamine synthetase repressor | 0.44 | NS |
| SACOL2043 | acetolactate synthase, large subunit, biosynthetic type | 0.40 | NS |
| SACOL0251 | 6-phospho-beta-glucosidase | 0.38 | 0.24 |
| SACOL0758**^#^ | 1-phosphofructokinase, FruB | 0.33 | 0.2 |
| SACOL2620 | 4-aminobutyrate aminotransferase | 0.33 | NS |
| SACOL0154 | aldehyde dehydrogenase | 0.31 | 0.46 |
| SACOL2042 | dihydroxy-acid dehydratase | 0.27 | NS |
| SACOL2583 | acetyltransferase, GNAT family | 0.22 | NS |
| SACOL1552 | Maltose operon repressor | NS | 3.04 |
| SACOL0707 | dihydroxyacetone kinase family protein | NS | 2.91 |
| SACOL2514 | gluconate transporter, permease protein | NS | 2.74 |
| SACOL2003 | phospholipase C | NS | 2.62 |
| SACOL1551 | alpha-glucosidase | NS | 2.60 |
| SACOL2516 | gluconate operon transcriptional repressor | NS | 2.51 |
| SACOL1622 | glycyl-tRNA synthetase | NS | 2.50 |
| SACOL1360 | aspartate kinase | NS | 2.50 |
| SACOL2597 | Hydrolase, alpha beta hydrolase family | NS | 2.43 |
| SACOL1074 | phosphoribosylaminoimidazole carboxylase, ATPase subunit | NS | 2.42 |
| SACOL1073 | phosphoribosylaminoimidazole carboxylase, catalytic subunit | NS | 2.40 |
| SACOL1428 | aspartokinase, alpha and beta subunits | NS | 2.34 |
| SACOL1216 | orotidine 5'-phosphate decarboxylase | NS | 2.33 |
| SACOL2154 | arginase | NS | 2.3 |
| SACOL1835 | Oxidoreductase, aldo/keto reductase family | NS | 2.2 |
| SACOL2618 | L-lactate dehydrogenase | NS | 2.17 |
| SACOL0308 | Carbohydrate kinase PfkB family | NS | 2.16 |
| SACOL0708 | DAK2 domain protein | NS | 2.11 |
| SACOL1783 | Acetyl-coenzyme synthetase | NS | 2.1 |
| SACOL1082 | phosphoribosylaminoimidazolecarboxamide formyltransferase/IMP cyclohydrolase | NS | 2.07 |
| SACOL2045 | ketol-acid reductoisomerase | NS | 2.06 |
| SACOL1075 | phosphoribosylaminoimidazole-succinocarboxamidesynthase | NS | 2.06 |
| SACOL1181 | ornithine carbamoyltransferase | NS | 2.04 |
| SACOL2185 | galactose-6-phosphate isomerase | NS | 2.02 |
| SACOL1430* | dihydrodipicolinate synthase, DapA | NS | 2.01 |
| SACOL1655 | 5’-methylthioadenosine nucleosidase | NS | 0.46 |
| SACOL0712 | lipase/esterase | NS | 0.46 |
| SACOL2198 | Alpha acetolactate decarboxylase | NS | 0.45 |
| SACOL1669 | O-methyltransferase family protein | NS | 0.45 |
| SACOL2634 | Anaerobic ribonucleoside triphosphate reductase activating protein | NS | 0.44 |
| SACOL1883 | CRC B family protein | NS | 0.44 |
| SACOL1729 | Threonyl-tRNA synthetase | NS | 0.44 |
| SACOL1593 | Glycine dehydrogenase subunit 2 | NS | 0.43 |
| SACOL0491 | Cobalamin synthesis protein, putative | NS | 0.43 |
| SACOL2635 | Anaerobic ribonucleoside triphosphate reductase | NS | 0.40 |
| SACOL1118 | GTP binding protein TypA | NS | 0.40 |
| SACOL1094 | Cytochrome d ubiqunol oxidase, subunit 1 | NS | 0.36 |
| SACOL1837 | S-adenosylmethionine synthetase | NS | 0.27 |
| SACOL0494 | NADH dehydrogenase subunit L | NS | 0.27 |
| **Cell envelope** | | | |
| SACOL0119 | cell wall surface anchor family protein | 2.01 | NS |
| SACOL0507 | LysM domain protein | 0.38 | NS |
| SACOL0486 | staphylococcus tandem lipoprotein | NS | 2.80 |
| SACOL0985 | surface protein, putative | NS | 2.51 |
| SACOL0247 | murein hydrolase regulator LrgA | NS | 2.04 |
| SACOL0479 | surface protein, putative | NS | 0.37 |
| **Unknown function** | | | |
| SACOL2571 | hypothetical protein | 10.53 | NS |
| SACOL0625 | hypothetical protein | 6.14 | NS |
| SACOL0624 | hypothetical protein | 5.07 | NS |
| SACOL1705 | hypothetical protein | 4.21 | NS |
| SACOL0157 | hypothetical protein | 2.83 | NS |
| SACOL1944 | hypothetical protein | 2.36 | NS |
| SACOL2733 | hypothetical protein | 2.35 | NS |
| SACOL2734 | hypothetical protein | 2.32 | 2.33 |
| SACOL0160 | hypothetical protein | 2.22 | NS |
| SACOL2315 | hypothetical protein | 2.13 | NS |
| SACOL1218 | hypothetical protein | 2.01 | 2.42 |
| SACOL0419 | hypothetical protein | 0.43 | 0.41 |
| SACOL0755 | hypothetical protein | 0.40 | NS |
| SACOL2557 | hypothetical protein | 0.39 | NS |
| SACOL0252 | hypothetical protein | 0.29 | 0.18 |
| SACOL1533 | hypothetical protein | NS | 2.74 |
| SACOL1532 | hypothetical protein | NS | 2.60 |
| SACOL2414 | hypothetical protein | NS | 2.59 |
| SACOL0421 | hypothetical protein | NS | 2.47 |
| SACOL0187 | hypothetical protein | NS | 2.4 |
| SACOL0854 | hypothetical protein | NS | 2.39 |
| SACOL2607 | hypothetical protein | NS | 2.36 |
| SACOL2709 | hypothetical protein | NS | 2.34 |
| SACOL0480 | hypothetical protein | NS | 2.27 |
| SACOL1033 | hypothetical protein | NS | 2.23 |
| SACOL0198 | hypothetical protein | NS | 2.2 |
| SACOL0076 | hypothetical protein | NS | 2.17 |
| SACOL0309 | hypothetical protein | NS | 2.14 |
| SACOL2481 | hypothetical protein | NS | 2.11 |
| SACOL0360 | hypothetical protein | NS | 2.09 |
| SACOL0487 | hypothetical protein | NS | 2.08 |
| SACOL0337 | hypothetical protein | NS | 2.07 |
| SACOL0488 | hypothetical protein | NS | 2.06 |
| SACOL2559 | hypothetical protein | NS | 2.03 |
| SACOL0355 | hypothetical protein | NS | 2.03 |
| SACOL0356 | hypothetical protein | NS | 2.02 |
| SACOL2373 | hypothetical protein | NS | 0.48 |
| SACOL2040 | hypothetical protein | NS | 0.48 |
| SACOL1275 | hypothetical protein | NS | 0.48 |
| SACOL1035 | hypothetical protein | NS | 0.48 |
| SACOL0769 | hypothetical protein | NS | 0.47 |
| SACOL2706 | hypothetical protein | NS | 0.46 |
| SACOL1903 | hypothetical protein | NS | 0.46 |
| SACOL0495** | hypothetical protein | NS | 0.44 |
| SACOL1643 | hypothetical protein | NS | 0.43 |
| SACOL2443 | hypothetical protein | NS | 0.41 |
| SACOL2082 | hypothetical protein | NS | 0.39 |
| SACOL2520 | hypothetical protein | NS | 0.36 |
