# Supplementary material for: Regulation of Hemolysin Expression and Virulence of Staphylococcus aureus by a Serine/Threonine Kinase and Phosphatase
Source: PLoS One. 2010 Jun 11;5(6):e11071. doi: 10.1371/journal.pone.0011071 (PMC2884019; doi:10.1371/journal.pone.0011071)
Supplement: Supplementary file 4 [file pone.0011071.s002.doc]

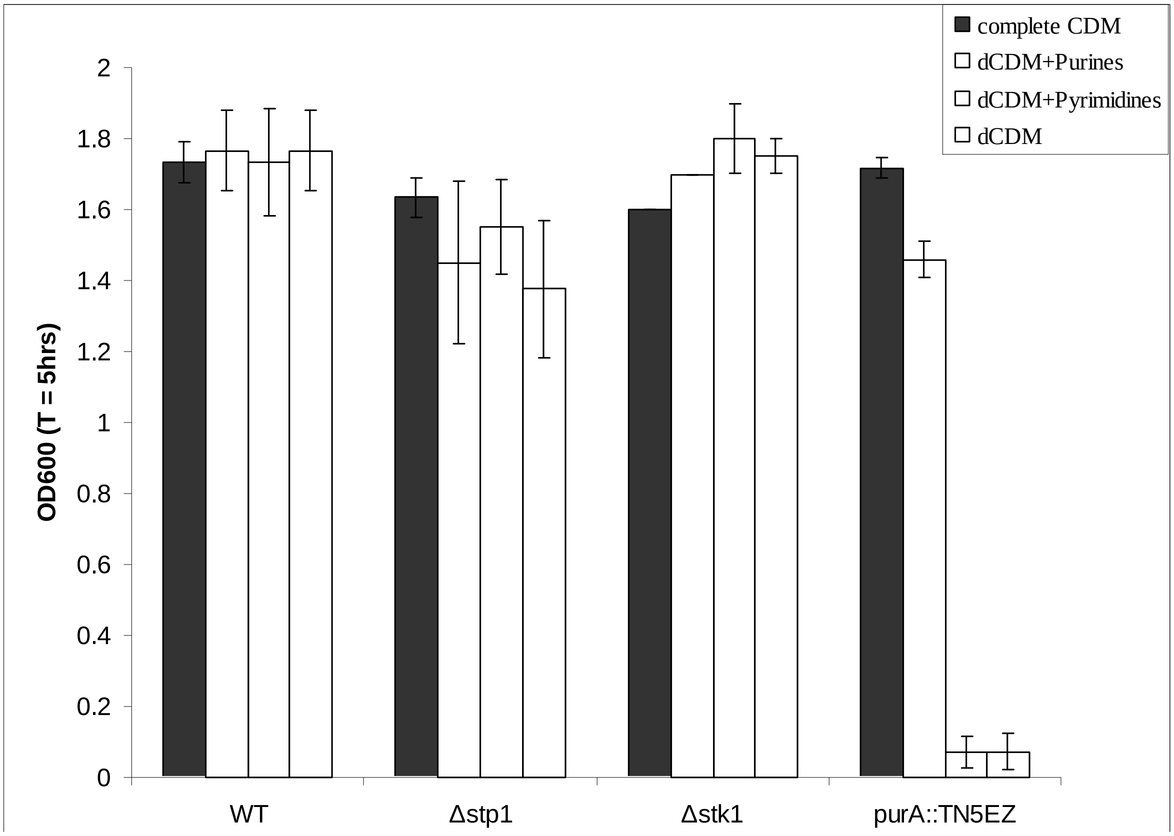
complete CDM
dCDM+Purines
dCDM+Pyrimidines
dCDM
OD600 (T = 5hrs)
2
1.8
1.6
1.4
1.2
1
0.8
0.6
0.4
0.2
0
WT
Δstp1
Δstk1
purA::TN5EZ
